# Eutypenoids A–C: Novel Pimarane Diterpenoids from the Arctic Fungus *Eutypella* sp. D-1

**DOI:** 10.3390/md14030044

**Published:** 2016-03-07

**Authors:** Liu-Qiang Zhang, Xiao-Chong Chen, Zhao-Qiang Chen, Gui-Min Wang, Shi-Guo Zhu, Yi-Fu Yang, Kai-Xian Chen, Xiao-Yu Liu, Yi-Ming Li

**Affiliations:** 1 04100217@163.comchen7673@163.comjusco105@163.comyangyifu@mail.shcnc.ac.cnkxchen@simm.ac.cn; 2 zqchen@simm.ac.cngmwang@simm.ac.cn; 3

**Keywords:** eutypenoid, novel pimarane diterpenes, *Eutypella*, Arctic fungi, immunosuppressive effect

## Abstract

Eutypenoids A–C (**1**–**3**), pimarane diterpenoid alkaloid and two ring A rearranged pimarane diterpenoids, were isolated from the culture of *Eutypella* sp. D-1 obtained from high-latitude soil of the Arctic. Their structures, including absolute configurations, were authenticated on the basis of the mass spectroscopy (MS), nuclear magnetic resonance (NMR), X-ray crystallography, and electronic circular dichroism (ECD) analysis. The immunosuppressive effects of eutypenoids A–C (**1**–**3**) were studied using a ConA-induced splenocyte proliferation model, which suggested that **2** exhibited potent immunosuppressive activities.

## 1. Introduction

Marine-sourced fungi are, increasingly, a rich source of novel and bioactive compounds [1,2,3], but natural products from Arctic fungi are rarely studied. Arctic fungi are abundant and functionally important in the Arctic, where they drive mineral and energy cycles, and influence the occurrence of other organisms as mutualists, decomposers, and pathogens [4]. Meanwhile, Arctic fungi living in low temperatures, strong ultraviolet radiation, low nutrition, *etc.*, might have evolved specific physiological and biochemical pathways to produce structurally novel and biological active metabolites, which can provide the opportunity for the discovery of new natural medicines.

**Figure 1:**
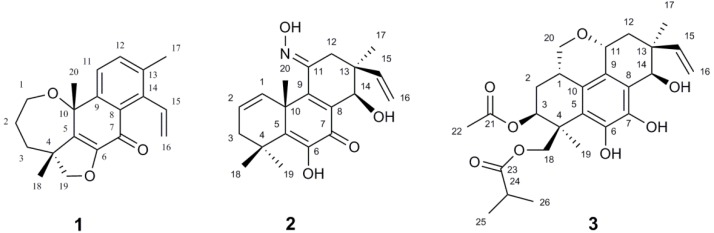
Structures of compounds **1**–**3**.

*Eutypella* (Diatrypaceae) species, from the South Sea in China and Thailand, have been widely investigated in recent years. Most of metabolites, including pimarane diterpenoids, cytochalasin derivatives, γ-lactones, sesquiterpenoids, polyketides, and cytosporin-related compounds, display moderate or significant cytotoxic and antimicrobial activities [5,6,7,8,9,10,11,12,13]. However, no such study has been carried out on *Eutypella* species from the Polar region. *Eutypella* sp. D-1 was isolated from high-latitude soil of the Arctic. Previously, we have reported six pimarane diterpenoids and four tyrosine-derived cytochalasins from the culture of *E.* sp. D-1 [14,15,16]. In the current study on the crude extract of *E.* sp. D-1, we reported the isolation, structure elucidation, and immunosuppressive effects of three novel pimarane diterpenoids (**1**–**3**) (Figure 1), each of which possesses a novel structure type of diterpenoid, respectively. Further, these compounds were evaluated for their immunosuppressive effects.

## 2. Results and Discussion

Under bioassay guidance, the chemical constituents of *E.* sp. D-1 have been extensively investigated further. In this paper, three new novel rearranged pimarane diterpenoids (**1**–**3**) were isolated from the EtOAc extract of its culture broth.

**Figure 2:**
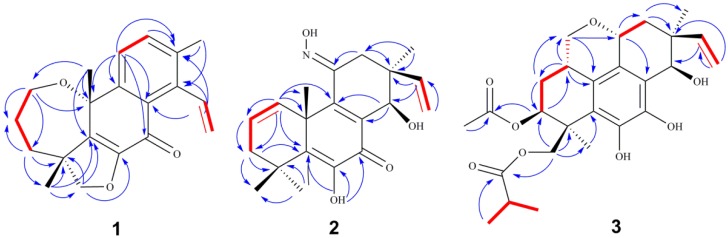
^1^H-^1^H COSY, HMBC correlations of eutyenoids A–C (**1**–**3**).

Eutypenoid A (**1**) was obtained as colorless needle crystals (EtOH). Its molecular formula C_20_H_22_O_3_ was determined on the basis of the high-resolution electron impact mass spectrometry (HREIMS) at *m*/*z* 310.1570 [M^+^] (calcd. 310.1569), corresponding to ten degrees of unsaturation. The ^1^H NMR spectrum of **1** showed a terminal vinyl group (*δ* 5.06, d, *J* = 18.0 Hz, 5.49, d, *J* = 11.5 Hz, 7.19, dd, *J* = 18.0, 11.5 Hz), two aromatic protons (*δ* 7.43, d, *J* = 8.0 Hz, 7.54, d, *J* = 8.0 Hz), and three methyl groups at *δ* 1.47, 1.60, 2.37 (each 3H, s). The ^13^C NMR spectrum showed 20 carbon resonances, which were assigned by distortionless enhancement by polarization transfer (DEPT) and heteronuclear single quantum coherence (HSQC) spectra to one lactone carbonyl carbon, ten olefinic carbons occupied six degrees of unsaturation. These data suggested that **1** is a diterpenoid possessing a tetracyclic ring system. Further structural information was derived by 2D NMR analysis, including HSQC, ^1^H ^1^H correlation spectroscopy (COSY), and HMBC (Figure 2). Correlations were detected for H-1/H-2/H-3 using COSY. The long range HMBC correlations of H-1/C-3 (*δ* 40.6) and C-10 (*δ* 76.9); H-2/C-4 (*δ* 49.8); H-3/C-1 (*δ* 67.2), C-5 (*δ* 138.6); H_3_-20/C-5 corroborated the oxepane skeleton of ring A. The furan ring was established using the HMBC correlations of H-19/C-3, C-5, C-18, and C-6. Additionally, the ^1^H-^1^H COSY correlations from H-11 to H-12 and the HMBC correlations from H-11/C-7, C-8, C-10 and C-13; H-12/C-9 and C-14 confirmed the structure of rings B and C. Correlations were detected for H-15/H-16 using ^1^H-^1^H COSY. The HMBC correlations from H-15/C-8, C-13; H_3_-17/C-12, C-13 and C-14, suggested that C-15 and C-17 were located at C-14 and C-13, respectively. Based on these data, a novel ring A rearrangement of the pimarane diterpenoid structure was determined and named eutypenoid A, with a new carbon skeleton.

**Figure 3:**
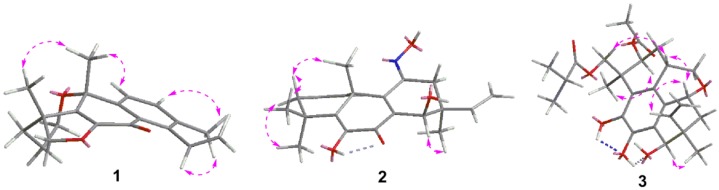
Key NOESY correlations of eutyenoids A–C (**1**–**3**).

**Figure 4:**
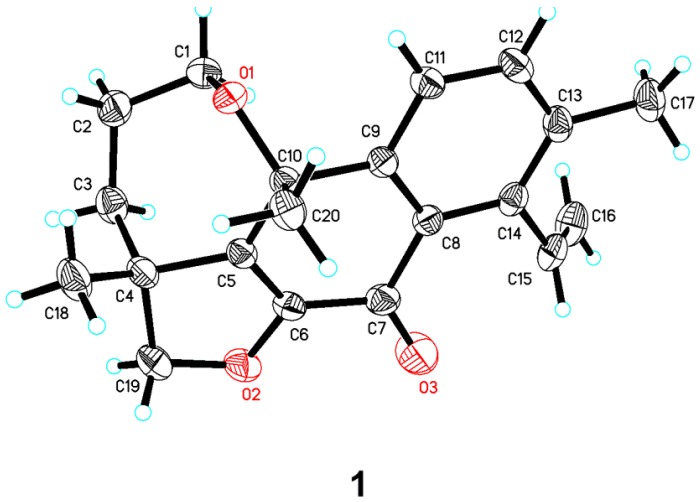
X-ray crystallographic structure of eutyenoid A (**1**).

The relative configuration of **1** was deduced from nuclear overhauser effect spectroscopy (NOESY) analysis (Figure 3). The correlations of H-18 with H-20 indicated β-orientations. Finally, the proposed structure of **1** was confirmed by X-ray crystallography analysis using anomalous scattering of Cu Kα radiation (Figure 4). Accordingly, the absolute configuration of 4*R*, 10*R* was established based on the value of the Flack absolute structure parameter −0.12 (10).

**Figure 5:**
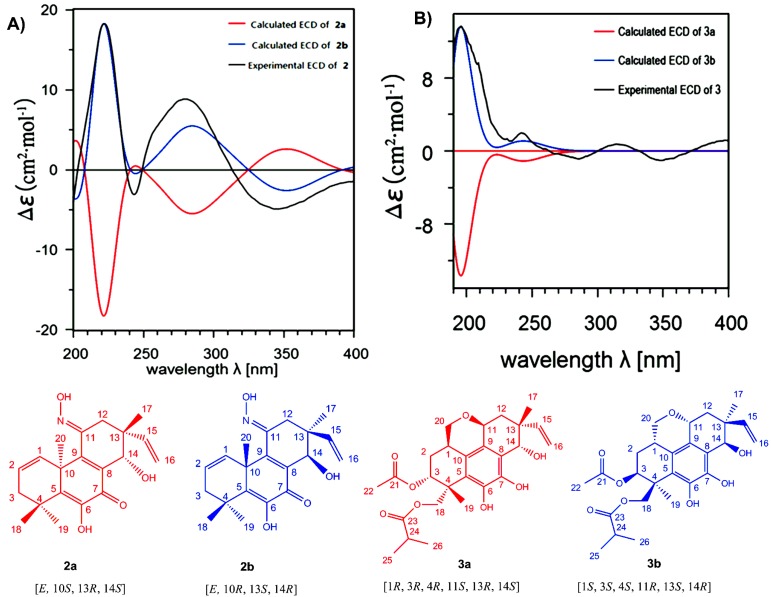
Comparison of experimental and calculated ECD spectra of **2** (**A**) and **3** (**B**). Geometries optimization were performed at theB3LYP/6-31G(d) level and ECD calculation were performed at the B3LYP/6-31G(d,p) level in methanol with the CPCM model.

Eutypenoid B (**2**) was isolated as a yellow powder. The HREIMS (*m*/*z* 343.1780) of **2** established a molecular formula of C_20_H_25_NO_4_ (calcd. 343.1784), with nine degrees of unsaturation. The ^1^H NMR spectrum of **2** showed signals assigned to two olefinic protons (*δ* 6.15, dd, *J* = 3.3, 9.5 Hz; 6.02, m), four methyl groups at *δ* 1.05, 1.28, 1.53, 1.62 (each 3H, s), and a terminal vinyl group (*δ* 5.20, d, *J* = 18.0 Hz, 5.17, d, *J* = 10.8 Hz; 6.06, dd, *J* = 18.0, 10.8 Hz), attached to a quaternary carbon, which is usually present in pimarane diterpenoids. The ^13^C NMR of **2** revealed 20 carbon signals. Interpretation of the ^1^H and ^13^C NMR (Table 1), and HSQC spectroscopic data showed the presence of a ketone carbon (*δ* 181.3), a ketoxime carbon (*δ* 153.5), eight olefinic carbons, including one methylene and three methine, three *sp^3^* quaternary carbons, one oxymethine carbon, two methylene carbons, and four methyl carbons. Specifically, the HSQC did not provide the correction between the carbons and protons (*δ* 6.76, s; 7.94, s), which were indicative of two active hydrogens. The planar structure of **2** was established by extensive analyses of its ^1^H-^1^H COSY and HMBC spectra (Figure 2). Key HMBC correlations from H_3_-18, 19 to C-3, 5, from H_3_-20 to C-1, 5, 9, 10, 11, from H-15, H_3_-17 to C-12, 13, 14, allowed the structure of **2** to be assigned as a pimarane diterpenoid. Additionally, HMBC corrections from 6-OH to C-5, 6, 7, from N-OH to C-11 established the enol moiety at C-6 and ketoxime moiety at C-11. ^1^H-^1^H COSY spectrum of **2** revealed the partial structure H-1 to H-3 via H-2, and the connectivity between H-15 and H-16. The relative configuration of **2** was established from the nuclear overhauser effect (NOE) effects observed in the NOESY experiment (Figure 3). The NOE correlation revealed the relative configuration of **2** partially, in which the cross peaks of H-14/ H_3_-17 indicated that H-14 and H_3_-17 possessed the same orientation. The absolute configuration of **2** was established by comparison experimental and calculated electronic circular dichroism (ECD) spectra using the time-dependent density functional theory (TD-DFT) method at the B3LYP/6-31G (d,p) level in methanol with the conductor-like polarizable continuum model (CPCM), the *E*-isomer of ketoxime possessed the lower energy. The overall pattern of calculated ECD spectrum of (*E*,10*R*,13*S*,14*R*)-**2b** was in accordance with the experimental data of **2** (Figure 5A). Therefore, the absolute configuration of **2** was established as *E*, 10*R*, 13*S*, 14*R*.

Eutypenoid C (**3**) was afforded as a yellow powder. The molecular formula was determined to be C_26_H_34_O_8_ with 10 degrees of unsaturation, based on HRESIMS (*m*/*z* 497.2143 [M + Na]^+^, calcd. for C_26_H_34_O_8_Na, 497.2146). NMR spectroscopic data of **3** (Table 1) showed the presence of the acetyl (*δ* 170.6 (d), 21.4 (s)) and an isobutyryl (*δ* 177.1 (d), 34.4 (t), 19.2 (s), 19.1 (s)). In addition to these two substituents, the ^1^H NMR spectrum of **3** also exhibited the characteristic pattern for a terminal vinyl group (*δ* 5.33, d, *J* = 17.7 Hz, 5.36, d, *J* = 10.7 Hz; 5.92, dd, *J* = 17.7/10.7 Hz), the ^13^C NMR spectrum of **3** in combination with HSQC data revealed two methyls and eight olefinic carbons. These data suggested that compound **3** is a disubstituted tetracyclic pimarane diterpenoid. The basic carbon skeleton of **3** was established by comprehensive analysis of the 2D NMR spectroscopic data, particularly the ^1^H-^1^H COSY and HMBC correlations (Figure 2). Correlations were detected for H_2_-20/H-1/H-2/H-3 by COSY, indicating C-20 was attached to C-1, the HMBC correlations from H_2_-20 to C-1, C-2, C-10, C-11, and from H-11 to C-8, C-9, indicating a pyranoid ring was formed through C-20/C-1/C-10/C-9/C-11. The acetylated and isobutyrylated positions were determined to be C-3 and C-19 based on the HMBC correlations from H-3 to *δ* 170.6, and from H-19 to *δ* 177.1, respectively. The relative configuration of **3** was established using NOESY (Figure 3). The correlations of H-11 with H-15, of H-11 with H-1, of H-1 with H_2_-19 indicated *β*–orientations, the correlation of H-17 with H-14, of H-3 with H-18 indicated *α*–orientations. TD-DFT ECD calculations of **3a** and **3b** were performed (Figure 5B), followed by comparison of experimental and calculational ECD spectra. The best agreement occurred between the experimental ECD curve and the calculated one for (1*S*,3*S*,4*S*,11*R*,13*S*,14*R*)-**3b**, indicating the absolute configuration of **3** as 1*S*, 3*S*, 4*S*, 11*R*, 13*S*, 14*R.*

**Figure 6:**
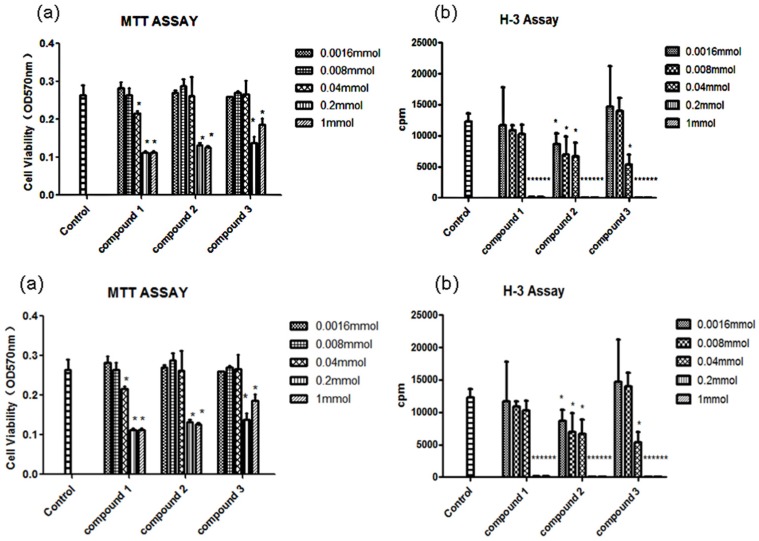
Cytotoxicity on splenocytes and inhibition on ConA-induced splenocyte proliferation of compounds **1**–**3**. (**a**) Cytotoxicity of compounds **1**–**3** on BALB/c mice splenocytes. The cells were incubated with different concentration of compounds **1**–**3** for 48 h. MTT was then added to the medium (0.5 mg/mL) and incubated for 4 h before the end of the incubation period. The cell viability was tested through the relative formazan concentration measured by the optical density at 570 nm (OD570 nm) using a microplate reader. (**b**) Inhibition of compounds **1**–**3** on ConA-induced splenocyte proliferation. BALB/c mice splenocytes (4 × 10^5^ cells/well) were stimulated by ConA (2 μg/mL) for 48 h in the presence of different compounds **1**–**3**. Cells were then pulsed with 0.25 μCi [^3^H]-thymidine 8 h before the end of the experiment and were assessed for [^3^H]-thymidine incorporation by counts per minute (cpm). Results are mean ± S.D. ^∗^ *p* < 0.05, ^∗∗^ *p* < 0.01, treatment group *versus* control.

Pimarane diterpenoids with anti-inflammtory effects have been reported [17,18]. In our bioassay of eutypenoids A–C with several anti-inflammatory models, we found that eutypenoid B (**2**) had an immunosuppressive effect. The immunosuppressive effects of eutypenoids A–C (**1**–**3**) were examined on splenocyte proliferation induced by concanavalin A (ConA) using a method described in the literature [19]. The results showed that compounds 1–3 had no cytotoxic effect on splenocytes at concentrations from 1.6 μmol to 40 μmol. Within the concentration range, eutypenoid B (**2**) exhibited significant inhibition of splenocyte proliferation under ConA induction, while eutypenoids A and C has no significant effects (Figure 6). Our findings, from an antiproliferation assay, propose that compound **2**, not only has no cytotoxic effect on splenocytes, but exhibited significant inhibition of splenocyte proliferation under ConA induction. Further study is needed to confirm the effect and pursue the mechanisms of action.

## 3. Experimental Section

### 3.1. General Experimental Procedures

Optical rotations were determined using a Perkin-Elmer 341 polarimeter. CD spectra were obtained on a Chirascan spectrometer (Applied Photophysics Ltd., Leatherhead, UK). The NMR spectra were recorded on a Bruker AM-400 spectrometer at 400 MHz for ^1^H and 100 MHz for ^13^C in CDCl_3_ or Bruker AVANCE-III instrument operating at 600 MHz for ^1^H and 150 MHz for ^13^C. ECD spectra were recorded in EtOH with a Chirascan CD spectrometer. ESIMS and HRESIMS were obtained using an Esquire 3000 plus and a Q-TOF-Ultima mass spectrometer, respectively. Silica gel (200 mesh to 300 mesh, Qingdao Haiyang Chemical Co., Ltd., Qingdao, China), C_18_ reversed phase silica gel (150 to 200 mesh, Fuji Silysia Chemical, Ltd., Aichi, Japan), MCI gel (CHP20P, 75 μm to 150 μm, Mitsubishi Chemical Industries, Ltd., Tokyo, Japan), and Sephadex LH-20 gel (Pharmacia Biotech AB, Uppsala, Sweden) were used for column chromatography (CC). High performance liquid chromatography was performed on an Angilent 1200 HPLC apparatus with an Eclipse XDB-C_18_ column (250 × 9.4 mm, 5 μm).

### 3.2. Fungal Strain

The fungus was isolated from the soil of London Island of Kongsfjorden of Ny-lesund District (altitude of 100 m) in the Arctic. It was isolated in potato dextrose agar (PDA) medium with incubation at 20 °C. Due to its chemical and morphological features, as well as the 18S rDNA (GenBank accession No. FJ430580), the strain was assigned to the genus *Eutypella*. The strain was deposited in PDA medium with the Second Military Medical University, Xiangyin Road 800, 200433, Shanghai, P. R. China. *Eutypella* sp. D-1 was cultured in potato dextrose broth (PDB; potato 1%, glucose 2%, dist. H_2_O 1000 mL).

### 3.3. Culture Condition

The fungus was maintained in PDA medium at 20 °C for 7 days, and then three pieces (0.5 × 0.5 cm^2^) of mycelial agar plugs were inoculated into 60 × 250 mL Erlenmeyer flasks, each containing 70 mL of PDB. After 5 d of incubation at 20 °C on a rotary shaker at 180 rpm, 70 mL of seed cultures were transferred into a total of 250 flasks (2.0 L) containing 700 mL of PDB. The liquid cultivation that followed was kept for 9 days at 20 °C and 180 rpm on a rotary shaker.

### 3.4. Extraction and Isolation

The culture (150 L) was centrifuged to give the broth and mycelia. The broth was exhaustively extracted with EtOAc three times, then the EtOAc layers were combined and evaporated under reduced pressure at a temperature not exceeding 40 °C to yield a dark brown gum (200 g), which was subjected to column chromatography on silica gel and eluted with EtOH in petroleum ether (PE) (0%–100%, stepwise) to yield 5 fractions (Fr. 1–Fr. 5). Fr. 3 (30 g) was subjected to CC by using EtOAc in PE (0%–100%, stepwise) to yield 4 fractions (Fr. 3A–Fr. 3D). Fr. 3A was further purified by Sephadex LH-20 using MeOH and then finally by semi-preparative HPLC (58:42 CH_3_CN-H_2_O; 3.0 mL/min) to give compound **2** (13 mg, *t*_R_ = 7.9 min). Fr. 3C was separated using CC of ODS with a MeOH gradient (10%–100%) in H_2_O to yield 12 fractions (Fr. 3CI-Fr. 3CXII). Fr. 3CVIII was separated by reversed-phase HPLC (50:50 CH_3_CN-H_2_O; 3.0 mL/min) to give compound **1** (3 mg, *t*_R_ = 17.0 min). Fr. 3CIX was separated by reversed-phase HPLC (58:42 CH_3_CN-H_2_O; 3.0 mL/min) to give compound **3** (4 mg, *t*_R_ = 27.1 min).

**Eutypenoid A** (**1**): colorless needle crystals; ${\left[\alpha \right]}_{D}^{25}$ = −8.0° (c 0.05, MeOH); CD (MeOH) Δε (nm): −9.6 (196), 5.3 (224), 9.0 (272), 3.8 (335), −4.2 (375); UV (MeOH) λ_max_: 319 nm, 266 nm, 217 nm; ^13^C and ^1^H NMR data (400 MHz; CDCl_3_), see Table 1; HREIMS *m*/*z* 310.1570 M^+^ (calcd. for C_20_H_22_O_3_, 310.1569).

A suitable colorless crystal (0.25 × 0.2 × 0.12 mm^3^) of **1** was grown by slow evaporation from an acetone solution. Diffraction intensity data were acquired with a Bruker APEX–II CCD area detector with graphite monochromated Cu K*α* radiation (λ = 1.54178 Å). Crystal data for **1**: C_20_H_22_O_3_ (formula weight 310.37), orthorhombic, space group, P2_1_2_1_2_1_ (#113), T = 296(2) K, a = 7.55310 (10) Å, b = 11.8579 (2) Å, c = 18.1930 (3) Å, V =1629.44 (4) Å^3^, D_c_ = 1.265 Mg/m^3^, Z = 4, F (000) = 664, *μ* (Cu K*α*) = 0.669 mm^−1^. A total of 9 465 reflections were collected in the range 4.451° < θ < 69.681°, with 2 900 independent reflections (*R*(int) = 0.0407); completeness to θ_max_ was 99.8%; semi empirical from equivalents 0.7533 and 0.6097; full matrix least squares refinement on F^2^; the number of data/restraints/parameters were 2900/0/212; goodness of fit on F^2^ = 1.044; final *R* indices [*I* > 2*σ*(*I*)], *R*_1_ = 0.0332 *wR*_2_ = 0.0907; *R* indices (all data), *R*_1_ = 0.0342, *wR*_2_ = 0.0918, largest difference peak and hole, 0.123 and −0.114 e/Å^3^. Flack parameter = −0.12 (10). Crystallographic data for the structure of 1 have been deposited at the Cambridge Crystallographic Data Centre under the reference number CCDC 1439456.

**Eutypenoid B** (**2**): yellow powder; ${\left[\alpha \right]}_{D}^{25}$ = −16.0° (c 0.05, MeOH); CD (MeOH) Δε (nm): 18.3 (222), −3.1 (243), 8.8 (280), −4.9 (346); UV (MeOH) λ_max_: 328 nm, 288 nm, 241 nm; ^13^C and ^1^H NMR data (400 MHz; CDCl_3_), see Table 1; HREIMS *m*/*z* 343.1780 M^+^ (calcd. for C_20_H_25_NO_4_, 343.1784).

**Eutypenoid C** (**3**): yellow powder; ${\left[\alpha \right]}_{D}^{25}$ = −12.0° (c 0.05, MeOH); CD (MeOH) Δε (nm): 13.6 (196), 2.0 (242), −0.8 (286), 0.7 (314), 1.0 (346); UV (MeOH) λ_max_: 288 nm, 235 nm; ^13^C and ^1^H NMR data (400 MHz; CDCl_3_), see Table 1; HRESIMS *m*/*z* 497.2143 [M + Na]^+^ (calcd. for C_26_H_34_O_8_Na, 497.2146).

### 3.5. Material and Method

Electronic circular dichroism (ECD) spectrum, associated *ab initio* (TD) DFT calculations, is a reliable spectroscopic tool for determining the absolute configuration of chiral compounds [20,21]. Firstly, conformation searches were carried out using a conformational search module in Schrodinger, with the OPLS_2005 Force field and the torsional sampling (MCMM) method. Then, the conformers were optimized using the DFT calculation. Frequency calculations were also performed to confirm that the geometries obtained correspond to energetic minima. The geometries of the optimized conformers were provided in the Supporting Information. Calculation of ECD spectra were performed using the TDDFT calculation. The ECD spectra were obtained by weighing the Boltzmann distribution rate of each conformer with the software SpecDis1.62 [22].

### 3.6. Antiproliferation Assay

Cell viability was assessed by performing an MTT assay [17]. In brief, splenocytes (4 × 10^5^ cells/well) were cultured in triplicate with or without compounds **1**–**3** in a 96 well plate at 37 °C in a 5% CO_2_ atmosphere for 48 h. MTT was then added to the medium (0.5 mg/mL) and incubated for 4 h before the end of the incubation period. The medium was removed, and the cells were diluted in dimethyl sulphoxide. The relative formazan concentration was measured by the optical density at 570 nm (OD570 nm) using a microplate reader (BioTek, PowWave XS2, Winooski, VT, USA). Compounds **1**–**3** did not react with MTT. Splenic lymphocytes (4 × 10^5^ cells/well) and compounds **1**–**3** in 96 well plates were cultured in triplicate for 48 h by using ConA (2 μg/mL). The cells were pulsed at 0.25 μCi/well of [^3^H]-thymidine for 8 h before the end of the culture period, and then harvested onto glass fiber filters. [^3^H]-thymidine incorporation was measured by counts per minute (cpm) using a beta scintillation counter (MicroBeta Trilux, PerkinElmer Life Sciences, Boston, MA, USA).

## 4. Conclusions

In conclusion, through the chemical investigation of *Eutypella* sp. D-1 from the Arctic, Eutypenoid B (**2**) a pimarane diterpenoid alkaloid was firstly isolated from natural metabolites, Eutypenoids A and C (**1** and **3**) were two unusual ring A rearranged pimarane diterpenoids with a new carbon skeleton, respectively. Among of them, Eutypenoid B (**2**) exhibited potent immunosuppressive activities.

## Figures and Tables

**Table 1:** ^1^H and ^13^C NMR data of eutypenoids A–C (**1**–**3**) in CDCl_3_.

No.	1		2		3	
	*δ*_C_^a^	*δ*_H_^b^ (*J* in Hz)	*δ*_C_^c^	*δ*_H_^d^ (*J* in Hz)	*δ*_C_^a^	*δ*_H_^b^ (*J* in Hz)
1	67.2, CH_2_	3.71, d (13.1)	132.2, CH	6.15, dd (3.3, 9.5)	28.7, CH	2.88, m
		2.90, t (12.3)				
2	27.6, CH_2_	1.97, m	127.8, CH	6.02, m	25.7, CH_2_	2.15, m 1.45, m
		1.58, m				
3	40.6, CH_2_	1.66, m	39.6, CH_2_	2.47, d (16.0)	72.8, CH	5.20, d (2.7)
				2.05, dd (7.7, 16.0)		
4	49.8, C		38.3, C		41.3, C	
5	138.6, C		146.6, C		122.7, C	
6	149.3, C		143.0, C		143.3, C	
7	179.0, C		181.3, C		142.3, C	
8	129.6, C		133.6, C		119.2, C	
9	148.8, C		153.0, C		124.7, C	
10	76.9, C		44.8, C		126.2, C	
11	124.1, CH	7.54, d (8.0)	153.5, C		66.5, CH	4.60, dd (6.2, 10.6)
12	135.0, CH	7.43, d (8.0)	28.7, CH_2_	2.86, s	42.6, CH_2_	2.18, m 1.72, m
13	136.5, C		39.7, C		43.3, C	
14	140.6, C		69.8, CH	4.68, s	76.5, CH	4.82, s
15	137.1, CH	7.19, dd (11.5, 18.0)	142.4, CH	6.06, dd (18.0, 10.8)	138.2, CH	5.92, dd (10.7, 17.7)
16	117.0, CH_2_	5.49, d (11.5)	114.4, CH_2_	5.20, d (18.0)	119.8, CH_2_	5.36, d (10.7)
		5.06, d (18.0)		5.17, d (10.8)		5.33, d (17.7)
17	21.4, CH_3_	2.37, s	23.9, CH_3_	1.05, s	23.5, CH_3_	1.31, s
18	20.1, CH_3_	1.47, s	27.9, CH_3_	1.28, s	66.3, CH_2_	5.16, d (10.7)
						4.31, d (10.7)
19	83.4, CH_2_	4.22, d (8.6)	27.4, CH_3_	1.53, s	22.1, CH_3_	1.45, s
		4.11, d (8.6)				
20	33.1, CH_3_	1.60, s	27.0, CH_3_	1.62, s	68.6, CH_2_	4.05, dd (6.5, 8.9)
						3.10, dd (8.9, 10.8)
6-OH/21				6.76, s	170.6, C	
N-OH/22				7.94, s	21.4, CH_3_	1.97, s
23					177.1, C	
24					34.4, CH	2.53, sep (7.0)
25					19.1, CH_3_	1.14, d (7.0)
26					19.2, CH_3_	1.12, d (7.0)

^a^ In CDCl_3_ (100 MHz); ^b^ In CDCl_3_ (400 MHz); ^c^ In CDCl_3_ (150 MHz); ^d^ In CDCl_3_ (600 MHz).

## Supplementary Files

Supplementary File 1
