# Supplementary material for: Eutypenoids A–C: Novel Pimarane Diterpenoids from the Arctic Fungus Eutypella sp. D-1
Source: Mar Drugs. 2016 Mar 7;14(3):44. doi: 10.3390/md14030044 (PMC4820298; doi:10.3390/md14030044)
Supplement: Supplementary File 1 [file marinedrugs-14-00044-s001.pdf]

Supplementary Materials: Eutypenoids A–C: Novel Pimarane Diterpenoids from the Arctic Fungus *Eutypella* sp. D-1

Liu-Qiang Zhang, Xiao-Chong Chen, Zhao-Qiang Chen, Gui-Min Wang, Shi-Guo Zhu, Yi-Fu Yang, Kai-Xian Chen, Xiao-Yu Liu and Yi-Ming Li

Conformer2a

Conformer 2a_1

C	2.28098000	-2.03466600	1.50371000
C	3.21644300	-0.88269200	1.72773100
C	3.44047000	-0.00016300	0.46425000
C	2.05702000	0.30665800	-0.13920500
C	1.09800500	-0.87700100	-0.39365000
C	1.34755000	-2.03668800	0.55305700
C	1.63938600	1.54029600	-0.52381100
C	0.21430900	1.85423000	-0.79526900
C	-0.77400100	0.80826900	-0.54597600
C	-0.37829000	-0.46293300	-0.31399200
C	-2.25059500	1.12171600	-0.50764800
C	-2.85266100	0.68294200	0.89421600
C	-2.23786500	-0.69564200	1.32748800
C	-1.48133700	-1.34674700	0.18836300
C	1.35787900	-1.37375600	-1.86996500
C	-2.48351700	1.75711500	1.93797400
C	-4.36442500	0.64422100	0.75001300
C	-5.12429500	-0.41538900	0.45274700
O	2.41540400	2.65186500	-0.59797700
O	-0.08066300	3.01844200	-1.09565900
O	-2.82780200	0.40600900	-1.59743300
C	4.20521400	1.25609800	0.93478900
C	4.36375900	-0.76282300	-0.52053900
N	-1.91752300	-2.47840500	-0.22573200
O	-1.33487700	-2.95359100	-1.41554100
H	2.38712300	-2.90710000	2.14662500
H	4.19282300	-1.25550900	2.06541300
H	2.84033400	-0.25197100	2.54793200
H	0.70915700	-2.90386000	0.40593900
H	-2.40172400	2.20068400	-0.63385300
H	-3.00492200	-1.38243400	1.68853100
H	-1.53844800	-0.52095400	2.15359700
H	2.42433400	-1.51359000	-2.04267400

H 0.86369600 -2.33243200 -2.03997100
H 0.98918900 -0.63493100 -2.58865200
H -1.39712800 1.87836600 2.01068800
H -2.91461000 2.73093200 1.67897000
H -2.85719400 1.47518800 2.92881600
H -4.85010100 1.61667800 0.85165200
H -6.20123400 -0.31658300 0.34406300
H -4.71355400 -1.41337500 0.32136600
H 1.78211000 3.36292000 -0.84994100
H -3.78778700 0.36611100 -1.43853800
H 3.59248000 1.88892800 1.58316300
H 4.55041000 1.86864200 0.10089300
H 5.08202300 0.92999600 1.50806400
H 4.01168700 -1.77801700 -0.72165400
H 4.45514600 -0.22619400 -1.47096800
H 5.36710500 -0.84387800 -0.08516300
H -1.17892300 -2.16884300 -1.97698200

Conformer 2a_2

C -2.78639100 1.99794300 1.11839000
C -3.87173400 1.30060700 0.34986900
C -3.54923300 -0.22102800 0.18418300
C -2.04705900 -0.46347700 -0.04604400
C -1.12378400 0.74034400 -0.26306000
C -1.50271700 1.72402800 0.85632300
C -1.51642200 -1.70619600 -0.06277800
C -0.06979700 -1.93333000 -0.08502100
C 0.85562200 -0.79831900 -0.05705800
C 0.38342800 0.47206700 -0.21405300
C 2.31569100 -1.22084100 0.06744000
C 3.26898000 -0.02426800 0.28816100
C 2.81240700 1.06691800 -0.70667800
C 1.39141800 1.55366200 -0.44602400
C -1.40907400 1.26601200 -1.71941600
C 3.15073300 0.46807700 1.74059800
C 4.68977600 -0.42695400 -0.06190700
C 5.76596900 -0.33019400 0.71982100
O -2.23844700 -2.85644300 0.00441100
O 0.32994400 -3.11418300 -0.10611900
O 2.69254200 -1.92953600 -1.11415200
C -3.97115000 -0.91434200 1.50742500
C -4.38499400 -0.79317600 -0.98326100

N 1.34057100 2.84843800 -0.54451100
O 0.20169300 3.60559800 -0.42222200
H -3.04648000 2.66894200 1.93399400
H -4.01419200 1.77219900 -0.63049100
H -4.83339300 1.39854500 0.86438100
H -0.71782800 2.09818300 1.51079600
H 2.38860900 -1.89566300 0.93303500
H 2.85932400 0.64460100 -1.71958900
H 3.47719400 1.93237400 -0.67587000
H -2.45794900 1.52574400 -1.86307800
H -0.80990900 2.14546400 -1.96154200
H -1.15713500 0.47182700 -2.42843300
H 3.74841100 1.37279100 1.89236600
H 2.11632900 0.70816100 2.00380400
H 3.50362000 -0.29592300 2.44155400
H 4.80624800 -0.82019500 -1.06851700
H 6.74484000 -0.63093900 0.35572300
H 5.73118600 0.04471800 1.73879200
H -1.55651400 -3.56597300 -0.01239200
H 2.16044300 -2.74482700 -1.09588600
H -5.04783200 -0.77881800 1.66623000
H -3.44251800 -0.47529600 2.36074200
H -3.75973300 -1.98403800 1.48371700
H -4.11193900 -0.32724200 -1.93656900
H -4.24114000 -1.87236700 -1.07586500
H -5.45084000 -0.60152700 -0.80740800
H -0.56867000 3.08150500 -0.11471700

Conformer 2a_3

C -2.80253100 2.02224400 1.04049900
C -3.87119400 1.30612800 0.26461200
C -3.53785600 -0.20889000 0.11365500
C -2.02984700 -0.44760600 -0.09005900
C -1.09279900 0.75744800 -0.26895900
C -1.51502000 1.76514100 0.80657400
C -1.51975900 -1.69833800 -0.12373200
C -0.07962500 -1.95913100 -0.09831500
C 0.85928800 -0.82846100 -0.09700900
C 0.41215700 0.45807000 -0.17614500
C 2.31257100 -1.24676600 -0.03965900
C 3.20613200 -0.14963600 0.59361000
C 2.92166600 1.11272000 -0.22223900

C 1.45280900 1.53779100 -0.21644000
C -1.32096400 1.26254900 -1.74344500
C 2.87095000 0.03765400 2.08909800
C 4.65003700 -0.60457000 0.49932600
C 5.61084700 -0.12737100 -0.29584200
O -2.26119500 -2.83921000 -0.07310100
O 0.29318100 -3.14007700 -0.02698400
O 2.69257800 -1.52171800 -1.39010200
C -3.97437500 -0.89727900 1.43505800
C -4.35212900 -0.79183600 -1.06379200
N 1.39598700 2.82892200 -0.30011100
O 0.14479200 3.42867100 -0.43075000
H -3.08612800 2.73154300 1.81508800
H -4.84324200 1.39714000 0.76237300
H -4.00435300 1.76663300 -0.72374100
H -0.74046100 2.24090700 1.39487600
H 2.36947800 -2.16757500 0.55578200
H 3.19412000 0.90880400 -1.26461500
H 3.52005700 1.96195300 0.11666700
H -2.36436700 1.53331300 -1.91083000
H -0.70947700 2.13668700 -1.96014400
H -1.06212100 0.45519600 -2.43580100
H 1.83179300 0.34265000 2.24186400
H 3.02924400 -0.89470200 2.64432700
H 3.51805900 0.80388500 2.52988100
H 4.89687200 -1.43134900 1.16955700
H 6.61514900 -0.54219900 -0.27195900
H 5.44864900 0.69792200 -0.98325000
H -1.58174900 -3.55211100 -0.04312200
H 3.62247400 -1.80575300 -1.36638300
H -3.46748700 -0.43897200 2.29117000
H -3.74835200 -1.96426000 1.42658800
H -5.05585300 -0.77445800 1.57248300
H -4.06325500 -0.32895500 -2.01422400
H -4.20701700 -1.87106600 -1.15003800
H -5.42091800 -0.59817700 -0.90693600
H 0.37998500 4.36999500 -0.46212300

Conformer 2a_4

C -2.89388000 1.90844100 1.06476800
C -3.92162600 1.15497900 0.27044200
C -3.52010800 -0.34993700 0.12393400

C	-2.00232600	-0.51891000	-0.06378000
C	-1.13351200	0.72778300	-0.26246200
C	-1.59148500	1.69663400	0.84006000
C	-1.41074200	-1.73388600	-0.05829200
C	0.04517700	-1.88939100	-0.04534100
C	0.91308900	-0.71067000	0.00476900
C	0.38314100	0.53443200	-0.17114900
C	2.39250000	-1.06404200	0.14052600
C	3.25998100	0.17504000	0.40982100
C	2.79313700	1.24942400	-0.59378400
C	1.34162900	1.66527600	-0.38208300
C	-1.40272200	1.23143300	-1.72983000
C	3.03722500	0.64926100	1.86935700
C	4.75563200	-0.04061900	0.26146900
C	5.43519800	-1.16820200	0.05401000
O	-2.07681600	-2.91818300	-0.00423600
O	0.50318200	-3.04906600	-0.06047300
O	2.80018800	-1.71500900	-1.06564200
C	-3.94327900	-1.05623900	1.43983900
C	-4.29459900	-0.96850200	-1.06192100
N	1.22823600	2.95527000	-0.48909700
O	0.04936100	3.65462400	-0.40490100
H	-3.20921000	2.56917700	1.86919700
H	-4.05841000	1.61378400	-0.71677800
H	-4.90126600	1.20824900	0.75675000
H	-0.84432500	2.11171900	1.51374400
H	2.48836600	-1.76459800	0.98310500
H	2.90364800	0.83469800	-1.60407300
H	3.40996400	2.14917400	-0.53411700
H	-0.84319200	2.13995800	-1.95950000
H	-1.08846600	0.44842200	-2.42624400
H	-2.45898000	1.43543500	-1.90626700
H	3.59330600	1.57437200	2.05728000
H	1.98335800	0.84641100	2.09041200
H	3.39806000	-0.10809500	2.57338400
H	5.32115100	0.88267300	0.40694300
H	6.52223000	-1.15728900	0.03086300
H	4.94782100	-2.11925800	-0.12433700
H	-1.36064800	-3.59331100	-0.00469200
H	2.29420700	-2.54727500	-1.07876300
H	-3.45948000	-0.58777800	2.30407700
H	-5.02912700	-0.97235600	1.56929700

H -3.67958000 -2.11448900 1.42826900
H -4.01735100 -0.49579200 -2.01063500
H -4.09716100 -2.04014100 -1.14257800
H -5.37286800 -0.82711300 -0.91708100
H -0.70158500 3.09378000 -0.11468000

Conformer3a

Conformer 3a_1

C -1.10282900 -1.82235600 -1.57862900
C -1.99146300 -0.86902000 -0.80121100
C -1.53223800 0.59759200 -0.88467900
C -0.02147600 0.69996200 -0.67892500
C 0.80085100 -0.43681100 -0.68027200
C 0.30240900 -1.82889900 -0.98658100
C 0.60562400 1.94036500 -0.53040100
C 1.98803700 2.04015500 -0.33699000
C 2.79047500 0.90433600 -0.27890200
C 2.17639200 -0.33577000 -0.46853700
C 4.29270000 1.04252900 -0.09655600
C 4.95497800 -0.26101400 0.40280000
C 4.45819900 -1.39980000 -0.50478900
C 2.95760200 -1.62834800 -0.42335300
C 6.47109900 -0.13029300 0.26777200
C 4.51933500 -0.48717200 1.83656100
C 5.30744200 -0.46728000 2.90733900
C -1.91360800 1.16381500 -2.26452300
C -2.28118500 1.38562300 0.20859700
O -3.69305800 1.17622100 0.02232100
C -4.43188200 0.68721100 1.02795700
C -5.88101100 0.53777200 0.61928100
O -3.99392900 0.42794000 2.12154500
C -6.50939900 1.92729700 0.45669000
C -6.01736200 -0.28882100 -0.66165000
O -1.94413400 -1.25742200 0.59011900
C -2.83403500 -2.16533300 1.01818900
C -2.64083800 -2.47146800 2.47320200
O -3.68071300 -2.64719500 0.30742700
O 2.62136400 -2.51757500 -1.49054100
O 4.62143100 2.14513400 0.75390200
C 1.28311500 -2.47140900 -1.96732400
O 2.46354700 3.31611100 -0.23583400
O -0.10918100 3.09898600 -0.59008300

H -1.53568100 -2.82498800 -1.55205900
H -1.07067200 -1.50802900 -2.62573300
H -3.02496900 -0.93706100 -1.14094300
H 0.30126800 -2.42439400 -0.06530200
H 4.73931400 1.29935000 -1.06345900
H 4.69748400 -1.15028100 -1.54447700
H 4.98499400 -2.32530500 -0.25839200
H 2.71151200 -2.14104100 0.51781300
H 6.73860700 -0.01227700 -0.78476700
H 6.96956300 -1.02407400 0.64868900
H 6.84324400 0.73779400 0.81429900
H 3.45197600 -0.63616800 1.99018900
H 4.89579600 -0.61982700 3.89832400
H 6.37850000 -0.31352800 2.83914700
H -1.61930000 2.21253200 -2.32193200
H -2.99298600 1.09330800 -2.41699600
H -1.40907700 0.62546100 -3.06822700
H -1.99236300 1.05377600 1.20284000
H -2.10831600 2.45181100 0.10046300
H -6.36296400 0.01764100 1.44934900
H -7.57042400 1.82463300 0.22230200
H -6.41543800 2.51732700 1.37056200
H -6.02541100 2.46746800 -0.35952100
H -7.07459200 -0.43829800 -0.88921200
H -5.54186600 -1.26567100 -0.55480100
H -5.55422300 0.23394500 -1.50127000
H -2.92892800 -1.58656700 3.04314000
H -3.26384900 -3.31667800 2.75380900
H -1.59095500 -2.68448700 2.67373400
H 4.49243400 1.86305800 1.67004200
H 0.98282000 -3.50102400 -2.17952800
H 1.25643800 -1.90212300 -2.90526300
H 3.34483900 3.27239200 0.17649500
H 0.50502200 3.82654400 -0.42056800

Conformer 3a_2

C 1.52586800 3.07520400 -0.40460100
C 2.52861500 1.93630500 -0.37155600
C 2.15435400 0.77008500 -1.30880800
C 0.67134000 0.43347700 -1.18627900
C -0.22139700 1.26378600 -0.49926700
C 0.16301400 2.59993900 0.08594200

C 0.15036900 -0.73307000 -1.74747900
C -1.19629900 -1.07713100 -1.60452700
C -2.06848900 -0.27570000 -0.87205900
C -1.56113900 0.91004100 -0.33735700
C -3.52050800 -0.67263600 -0.70266100
C -4.20678600 0.04721700 0.47783700
C -3.89552100 1.54869200 0.32924800
C -2.41503300 1.85293300 0.47474900
C -5.71988800 -0.15535800 0.38523200
C -3.63501900 -0.48473600 1.77601300
C -4.32761400 -0.83368700 2.85388400
C 2.48875900 1.17909500 -2.75560000
C 3.05045000 -0.44097900 -0.98009500
O 2.49312900 -1.17036400 0.12151700
C 2.59707300 -2.50738700 0.09738400
C 1.72464800 -3.14836700 1.15517600
O 3.29108300 -3.10875600 -0.68243400
C 2.07446700 -2.63135500 2.55207300
C 0.24787400 -2.89177600 0.83169100
O 2.56043300 1.47446400 0.99757600
C 3.74482600 1.27988500 1.59245400
C 3.59099500 0.81294000 3.01177700
O 4.80849800 1.45304800 1.05158400
O -2.23304000 3.21793700 0.09544800
O -3.56011900 -2.09558700 -0.53523400
C -0.92011200 3.61236400 -0.28695400
O -1.55032000 -2.25543800 -2.19991600
O 0.95831000 -1.59728400 -2.42284600
H 1.88852900 3.89780000 0.21561000
H 1.44680000 3.44938300 -1.42920700
H 3.52825800 2.28323700 -0.63569200
H 0.19117300 2.51347800 1.17884700
H -4.06279300 -0.41035300 -1.62163400
H -4.21270000 1.88562400 -0.66428300
H -4.46312500 2.11599500 1.07098800
H -2.12455200 1.75593000 1.53135300
H -6.08917400 0.20425300 -0.57813400
H -6.23324100 0.40398500 1.16967500
H -5.99823400 -1.20669300 0.49705000
H -2.55060300 -0.57913900 1.80504400
H -3.82230500 -1.19008500 3.74394500
H -5.40977900 -0.78188400 2.89445300

H 2.19804400 0.37972900 -3.43820700
H 3.56292000 1.35557200 -2.86454000
H 1.95874000 2.08662100 -3.04578600
H 3.12495700 -1.10569400 -1.83501300
H 4.05028000 -0.09644200 -0.70536700
H 1.93401600 -4.21797600 1.09342900
H 1.47908300 -3.15919800 3.29937700
H 3.13037600 -2.78397200 2.78607800
H 1.85129100 -1.56532700 2.62387000
H -0.38413500 -3.43976200 1.53368900
H -0.00639600 -3.21266100 -0.18162500
H 0.02258600 -1.82554200 0.91707000
H 3.93850400 -0.22021200 3.06790800
H 4.23328300 1.41865400 3.65050000
H 2.55829400 0.87017800 3.34586700
H -4.47362700 -2.39144400 -0.60469400
H -0.71576400 4.57391100 0.19258800
H -0.89948100 3.75735700 -1.37433100
H -2.36426600 -2.56807500 -1.76521700
H 0.42017800 -2.36010600 -2.67647800

Conformer 3a_3

C -1.67826900 2.72691800 0.36195800
C -2.55846700 1.52416100 0.66450600
C -1.95630900 0.58051200 1.72025600
C -0.46433300 0.37966400 1.47378700
C 0.23367700 1.10974400 0.50709700
C -0.36445200 2.26359500 0.25644900
C 0.24855900 -0.58689200 2.18307900
C 1.59664900 -0.84620100 1.91481700
C 2.26963300 -0.16122600 0.90283100
C 1.56779600 0.82904900 0.21230200
C 3.73526800 -0.40713900 0.61382700
C 4.11326600 -0.01169900 0.82851200
C 3.68134500 1.44741200 1.01944600
C 2.17600800 1.63196800 0.91151400
C 5.63733500 -0.12093100 0.99315200
C 3.45775300 -0.96648100 1.80694200
C 3.07989100 -0.67107600 3.04577200
C -2.20376500 1.19182700 3.11096800
C -2.71934000 -0.76680600 1.67424100
O -2.07332400 -1.67349200 0.77812200

C -2.71260500 -2.13370800 0.29974600
C -1.74216000 -2.85880600 1.21185800
O -3.89346500 -1.99257000 0.49997000
C -2.45251100 -3.41674600 2.43840000
C -0.57864800 -1.92827900 1.58581100
O -2.73107200 0.77182400 0.55802600
C -3.66273000 1.22123700 1.41753100
C -3.70120600 0.39801500 2.67086600
O -4.36918300 2.16883700 1.18540200
O 1.93675800 3.03352200 0.75843500
O 4.02710400 -1.78636300 0.87102600
C 0.65225900 3.40737300 0.27068700
O 2.16299200 -1.79987900 2.71258000
O -0.36340900 -1.31196300 3.15949800
H -2.20636100 3.40727300 0.30879000
H -1.48306500 3.26869800 1.29193600
H -3.54667900 1.84194000 1.00262100
H -0.54664300 1.95398700 1.29364200
H 4.33333000 0.20810000 1.29957700
H 4.14956400 2.05633700 0.23835200
H 4.03645900 1.83289200 1.97866900
H 1.69707600 1.30819800 1.84516400
H 6.15217600 0.48314700 0.23997500
H 5.92794600 0.23449400 1.98333900
H 5.97466300 -1.15648100 0.90477000
H 3.35992000 -1.98820200 1.44837500
H 2.66821700 -1.43113300 3.69955000
H 3.17040100 0.33005600 3.45602400
H -1.73489000 0.57065600 3.87423400
H -3.27686400 1.24598300 3.31618000
H -1.78919400 2.19802900 3.18324700
H -2.70327400 -1.23866800 2.65376600
H -3.75058700 -0.60617200 1.35674900
H -1.33739500 -3.68441300 0.61590300
H -1.75928500 -4.02020800 3.02686700
H -3.30338800 -4.03828300 2.15750500
H -2.81917300 -2.60661800 3.07340300
H 0.11252700 -2.45206300 2.24913500
H -0.03508900 -1.59243300 0.70041600
H -0.95023300 -1.04212900 2.10865800
H -4.01302300 -0.61508300 2.41310800
H -4.39964300 0.84284200 3.37480500

H -2.70342800 0.34626200 3.10908900
H 4.97652500 -1.88540500 0.99692800
H 0.28535300 4.22473100 0.89851100
H 0.76475500 3.78324500 0.75381800
H 2.97906700 -2.09852300 2.27465100
H 0.27116000 -1.97329900 3.46729800

Conformer 3a_4

C -0.92427100 -1.63139700 -1.90236400
C -1.86640500 -0.74138400 -1.11331300
C -1.40572700 0.72713000 -1.04493900
C 0.08747500 0.80703600 -0.73363800
C 0.91215500 -0.32641400 -0.77445800
C 0.44140400 -1.68763700 -1.22665300
C 0.69696300 2.02554700 -0.42676700
C 2.06265400 2.10803800 -0.12885700
C 2.86229300 0.96739300 -0.10675000
C 2.26576300 -0.24915700 -0.44676700
C 4.34697600 1.04877500 0.17984200
C 4.91385300 -0.29338300 0.68710300
C 4.53380100 -1.35439200 -0.35189900
C 3.03096600 -1.55201500 -0.46933900
C 6.44430300 -0.18810600 0.77675500
C 4.37340100 -0.58602800 2.07306100
C 4.16486700 -1.79271700 2.58680000
C -1.70612600 1.40419700 -2.39384500
C -2.22392100 1.42287000 0.06162400
O -3.61621900 1.14479600 -0.17816600
C -4.36201200 0.63994700 0.81575000
C -5.77236400 0.35715000 0.34807700
O -3.95272400 0.46128500 1.93594700
C -6.47324500 -0.59838000 1.30518700
C -6.52500100 1.68846900 0.21039100
O -1.91624500 -1.25747100 0.23731600
C -2.94948900 -2.04297800 0.58110500
C -2.80561600 -2.54236800 1.98779200
O -3.87341000 -2.28553800 -0.15488600
O 2.80415100 -2.28633600 -1.67471600
O 4.57088400 2.10371200 1.12324000
C 1.48089000 -2.25583400 -2.19567800
O 2.51538000 3.37308800 0.11307200
O -0.02062600 3.18434600 -0.42958700

H -1.35418000 -2.63272500 -1.98487300
H -0.82688100 -1.23591900 -2.91768800
H -2.87204200 -0.77441500 -1.52827000
H 0.38146600 -2.35320600 -0.35688700
H 4.86731300 1.30238000 -0.75374900
H 4.90506200 -1.03392200 -1.33138300
H 5.01675100 -2.30840100 -0.12515500
H 2.67299600 -2.16260900 0.36951500
H 6.86896000 0.11015800 -0.18649100
H 6.86288300 -1.15599100 1.05845000
H 6.75156200 0.53479000 1.53668000
H 4.21155900 0.29231200 2.69281000
H 3.82657700 -1.91196500 3.60932300
H 4.32581100 -2.70239000 2.01646700
H -1.38120600 2.44476100 -2.36162900
H -2.77913000 1.37777300 -2.59614300
H -1.18267700 0.90969800 -3.21330600
H -1.94516000 1.05454300 1.04617000
H -2.09807700 2.50068400 0.01681900
H -5.68370500 -0.10122200 -0.64059700
H -7.48398000 -0.80512300 0.94842700
H -5.92908500 -1.54152200 1.37726400
H -6.54120300 -0.15923700 2.30238500
H -7.53416800 1.50399800 -0.16154900
H -6.01792100 2.36135500 -0.48224600
H -6.60466500 2.18178300 1.18261400
H -2.89066100 -1.68932300 2.66272900
H -3.59000900 -3.26472500 2.19881100
H -1.82259600 -2.99362900 2.12348000
H 5.49968800 2.35537300 1.09091700
H 1.21104400 -3.27937100 -2.47203700
H 1.47764100 -1.64105200 -3.10428000
H 3.37084000 3.29417800 0.57072400
H 0.57616700 3.89373200 -0.15438000

Conformer 3a_5

C -1.02002300 -1.87089100 -1.57535600
C -1.94173800 -1.01502500 -0.72921600
C -1.53708700 0.47334800 -0.69589700
C -0.02286200 0.62630200 -0.56196600
C 0.84564700 -0.47090300 -0.64173700
C 0.38865300 -1.86555800 -0.99507900

C 0.55288900 1.88218300 -0.35547400
C 1.93515900 2.03548500 -0.19865600
C 2.78799700 0.93393800 -0.22190400
C 2.22123200 -0.32111700 -0.45981200
C 4.28606500 1.11756700 -0.07296300
C 5.01453700 -0.17577200 0.34825500
C 4.53983800 -1.28472000 -0.60906000
C 3.05628600 -1.58035100 -0.47458600
C 6.52311400 0.01051200 0.18054500
C 4.63355400 -0.51566200 1.77410900
C 5.46000200 -0.92451700 2.72962600
C -2.03490100 1.13765400 -1.99101700
C -2.22762100 1.12334000 0.52172600
O -3.59275100 0.67589900 0.59238500
C -4.57438700 1.52976100 0.25878800
C -5.92745500 0.85669400 0.30819600
O -4.38460900 2.67238300 -0.07231000
C -6.04793400 -0.08615900 -0.89737000
C -6.14748100 0.09717200 1.61741400
O -1.90132300 -1.51646400 0.62322200
C -2.96247500 -2.20369900 1.07259500
C -2.86503400 -2.43257200 2.55219100
O -3.87499400 -2.56271900 0.37296000
O 2.71688900 -2.47400400 -1.53655200
O 4.50782000 2.16772100 0.87608000
C 1.37782800 -2.43904300 -2.00768300
O 2.35030200 3.32627200 -0.03988900
O -0.21928600 3.00374700 -0.31090600
H -1.41269100 -2.88935600 -1.62450200
H -1.00575000 -1.47544600 -2.59584700
H -2.96887300 -1.08619000 -1.08271900
H 0.40977600 -2.49578000 -0.09750400
H 4.68845200 1.43195100 -1.04599300
H 4.72972600 -0.97452400 -1.64304900
H 5.11177900 -2.19756700 -0.42444700
H 2.87484600 -2.11126600 0.47162000
H 6.75377700 0.31188200 -0.84410300
H 7.05096100 -0.92398500 0.37986800
H 6.91801500 0.76330900 0.86764300
H 3.57529200 -0.41699100 2.00977200
H 5.08689300 -1.16578600 3.71807000
H 6.52674300 -1.03419700 2.56954700

H -1.77223500 2.19533000 -1.99310800

H -3.12198300 1.04866900 -2.07273000

H -1.58764700 0.66804800 -2.86866500

H -1.75793700 0.79024600 1.44569800

H -2.20261700 2.20545600 0.45402400

H -6.65624300 1.66383100 0.21341800

H -7.05465300 -0.50697600 -0.93420800

H -5.86448200 0.43943600 -1.83731900

H -5.33422300 -0.90737400 -0.79913200

H -7.16739100 -0.29128000 1.64605600

H -6.00200200 0.74055800 2.48743900

H -5.45666700 -0.74545400 1.68299800

H -3.60245000 -3.16865400 2.86128500

H -1.86003500 -2.75338300 2.82340700

H -3.06249700 -1.48196500 3.05417300

H 5.42790300 2.44543500 0.82015100

H 1.10484000 -3.46635200 -2.26249300

H 1.33080500 -1.83129900 -2.92070200

H 3.23485800 3.30017000 0.36636100

H 0.36649200 3.74902800 -0.11893700

Conformer 3a_6

C 1.36482700 3.11921000 -0.44245500

C 2.39857800 2.00889500 -0.45335100

C 2.01425500 0.82689800 -1.36490200

C 0.55387300 0.43610100 -1.15504100

C -0.33655700 1.25641000 -0.45034300

C 0.03577600 2.60977800 0.10369300

C 0.04777000 -0.75872400 -1.66904400

C -1.28448200 -1.13309900 -1.47295400

C -2.15570000 -0.33153700 -0.74063600

C -1.66329400 0.87682200 -0.24327600

C -3.60817600 -0.74052300 -0.55656200

C -4.29548000 -0.01899500 0.61429100

C -3.99980200 1.48401300 0.46591600

C -2.51858000 1.80316800 0.58820700

C -5.80982600 -0.25086300 0.52046800

C -3.82194700 -0.49267200 1.97546600

C -2.74683900 -1.21753500 2.27536500

C 2.24173600 1.24280100 -2.83035500

C 2.98332500 -0.34117100 -1.09379100

O 2.53081600 -1.09794100 0.03627600

C	2.72897400	-2.42409600	0.00505800
C	1.94999500	-3.12290600	1.09755100
O	3.43120400	-2.97417200	-0.80454900
C	2.34731200	-2.59984800	2.47974700
C	0.44814700	-2.93418500	0.85679300
O	2.50885500	1.55376300	0.91390800
C	3.72488700	1.39762500	1.45363900
C	3.65118500	0.94391500	2.88372800
O	4.75661700	1.59677800	0.86248100
O	-2.36590300	3.17775500	0.22669400
O	-3.76541200	-2.16225400	-0.47546600
C	-1.08701400	3.58747500	-0.23962200
O	-1.62994000	-2.33304600	-2.02925300
O	0.85394300	-1.61425700	-2.35850000
H	1.73074900	3.95071200	0.16388000
H	1.23403400	3.49343900	-1.46171300
H	3.37386000	2.38300200	-0.76697000
H	0.10556300	2.53764300	1.19601400
H	-4.14988000	-0.46469700	-1.46824900
H	-4.33915700	1.81685700	-0.52127400
H	-4.55986400	2.05005300	1.21534700
H	-2.20932200	1.69431600	1.63734200
H	-6.19740100	0.15001200	-0.41946000
H	-6.32241300	0.25059500	1.34426900
H	-6.04309700	-1.31624700	0.56479000
H	-4.46714800	-0.17184200	2.79229900
H	-2.53044300	-1.47999100	3.30435700
H	-2.03631000	-1.55899400	1.52913400
H	1.94423800	0.42756600	-3.49115200
H	3.29890500	1.46305400	-3.00542200
H	1.65709500	2.12523300	-3.09133400
H	3.03637100	-1.00066700	-1.95427600
H	3.98104200	0.05075000	-0.88131300
H	2.21014300	-4.17954100	1.01165500
H	1.82477800	-3.16649600	3.25266200
H	3.42156900	-2.69373500	2.65344000
H	2.06854500	-1.54887500	2.57584100
H	-0.11878300	-3.50404000	1.59626500
H	0.14782800	-3.26595600	-0.14048600
H	0.19002100	-1.87646400	0.95389500
H	4.07656400	-0.05907400	2.94595000
H	4.26741300	1.60647300	3.49142300

H 2.62862000 0.93381500 3.25183300

H -3.62017400 -2.43607900 0.43867400

H -0.88159300 4.56638000 0.20251000

H -1.12466800 3.70225900 -1.33054700

H -2.45935200 -2.62713200 -1.61121800

H 0.32318400 -2.38707200 -2.59643200

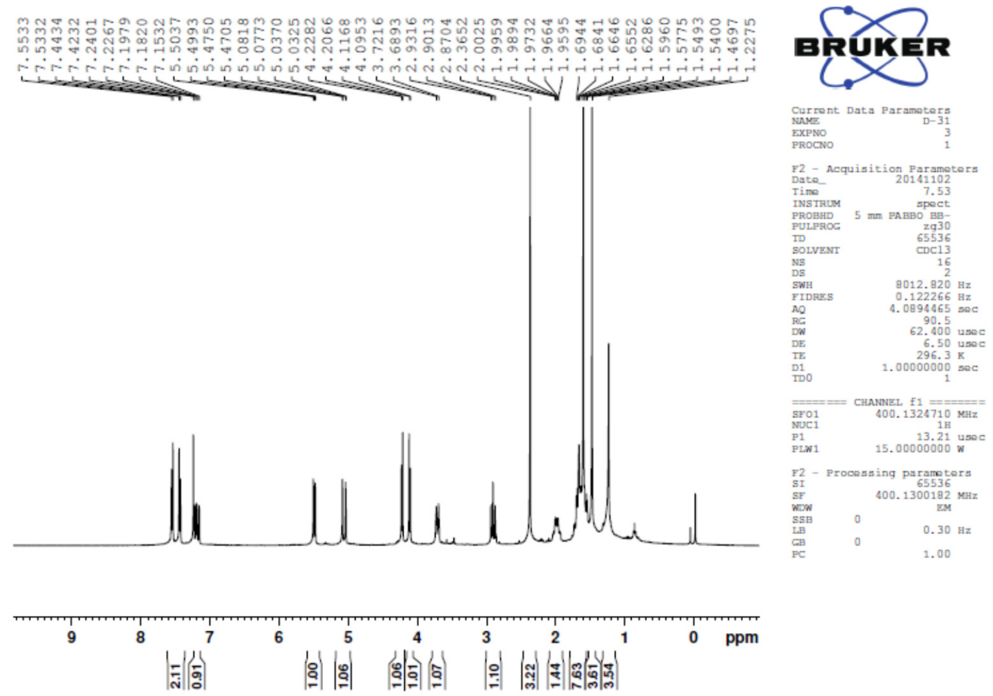

Figure S1. ^{1}H NMR ($CDCl_3$, 400 MHZ) spectrum of **1**.

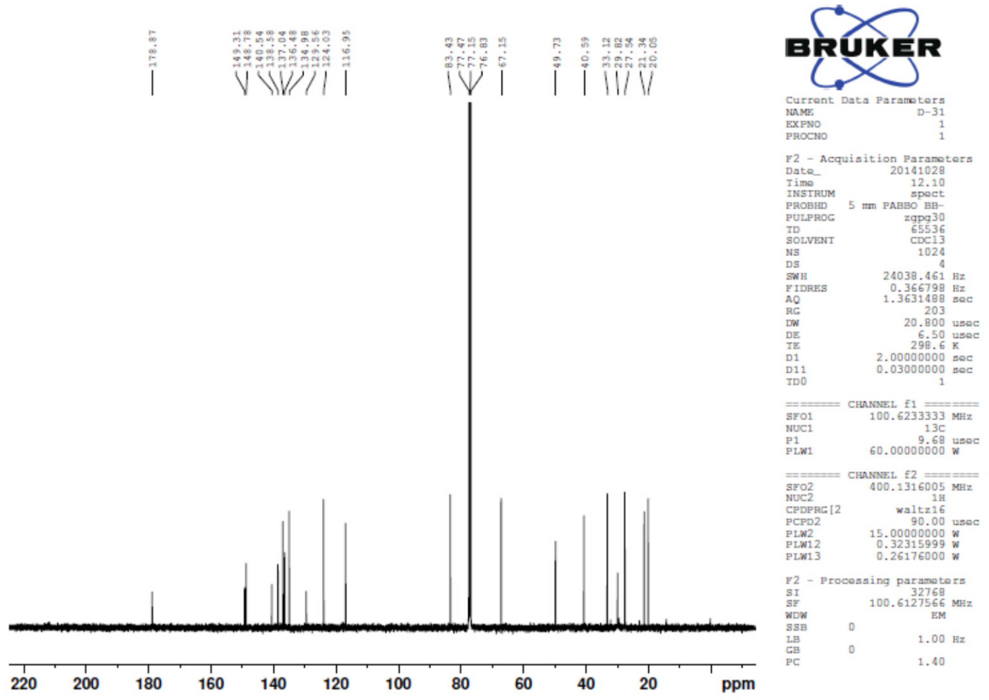

Figure S2. ^{13}C NMR ($CDCl_3$, 100 MHZ) spectrum of **1**.

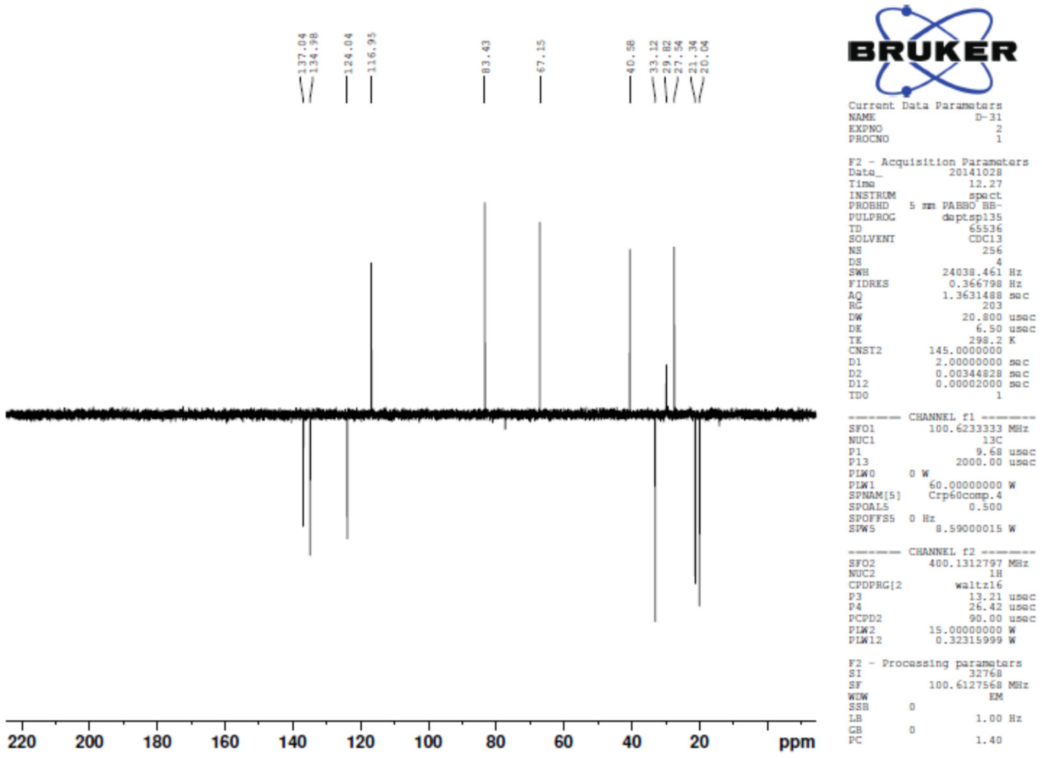

Figure S3. DEPT ($CDCl_3$, 100 MHZ) spectrum of **1**.

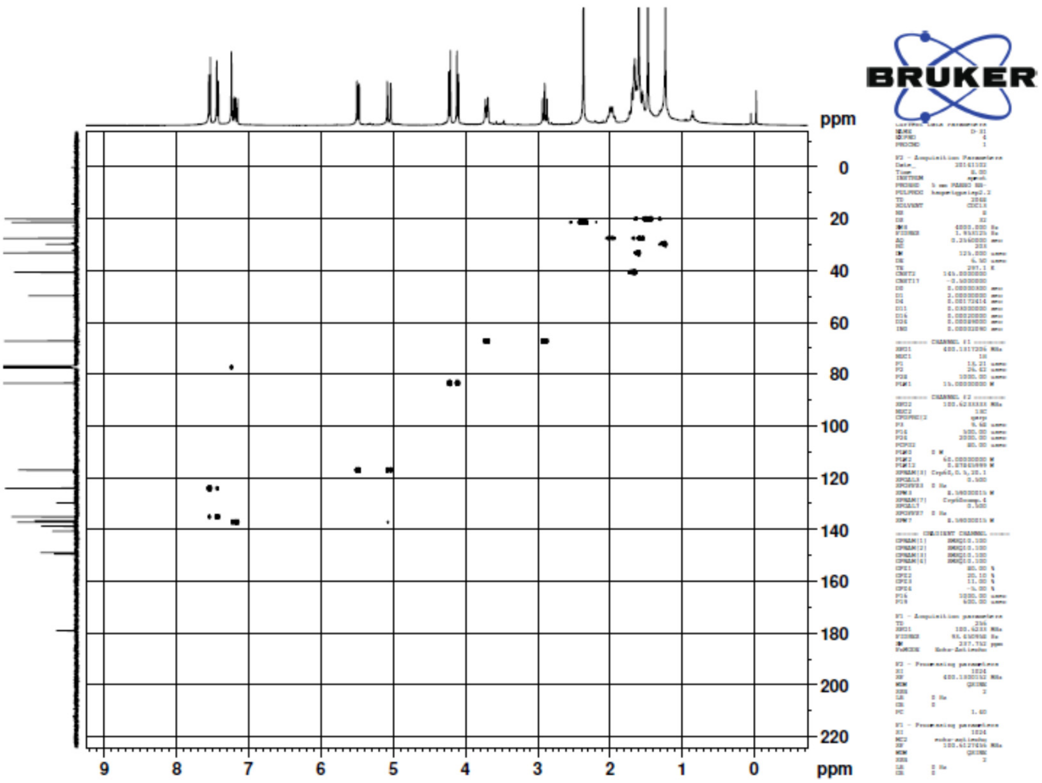

Figure S4. HSQC spectrum of **1**.

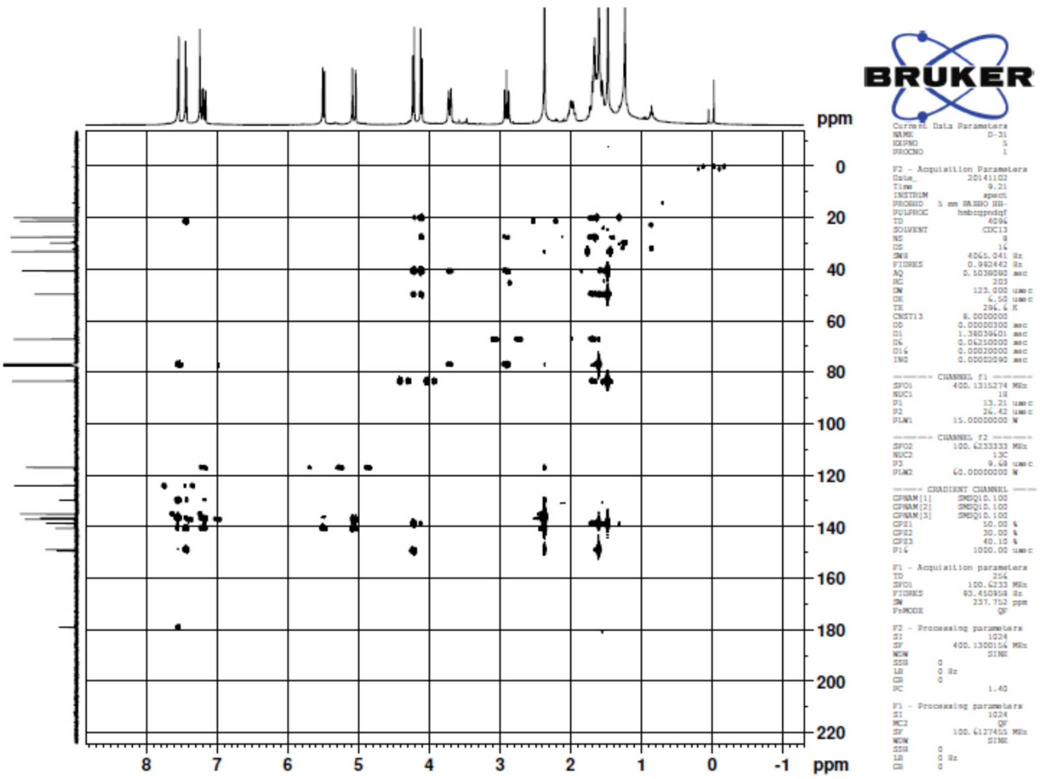

Figure S5. HMBC spectrum of **1**.

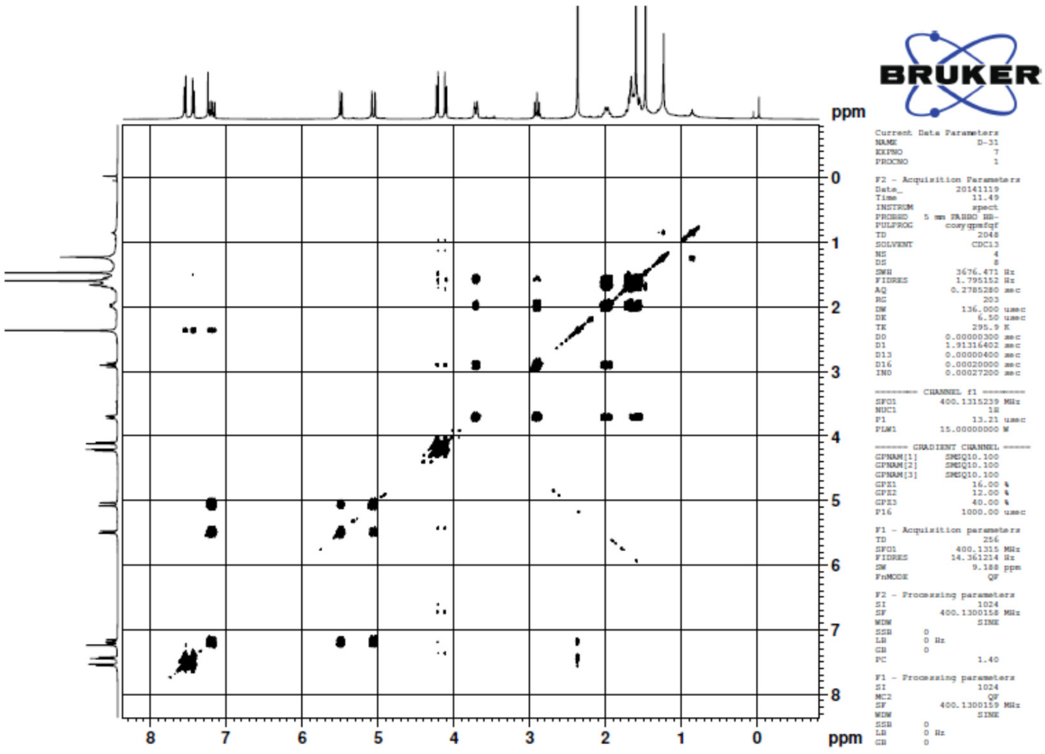

Figure S6. COSY spectrum of **1**.

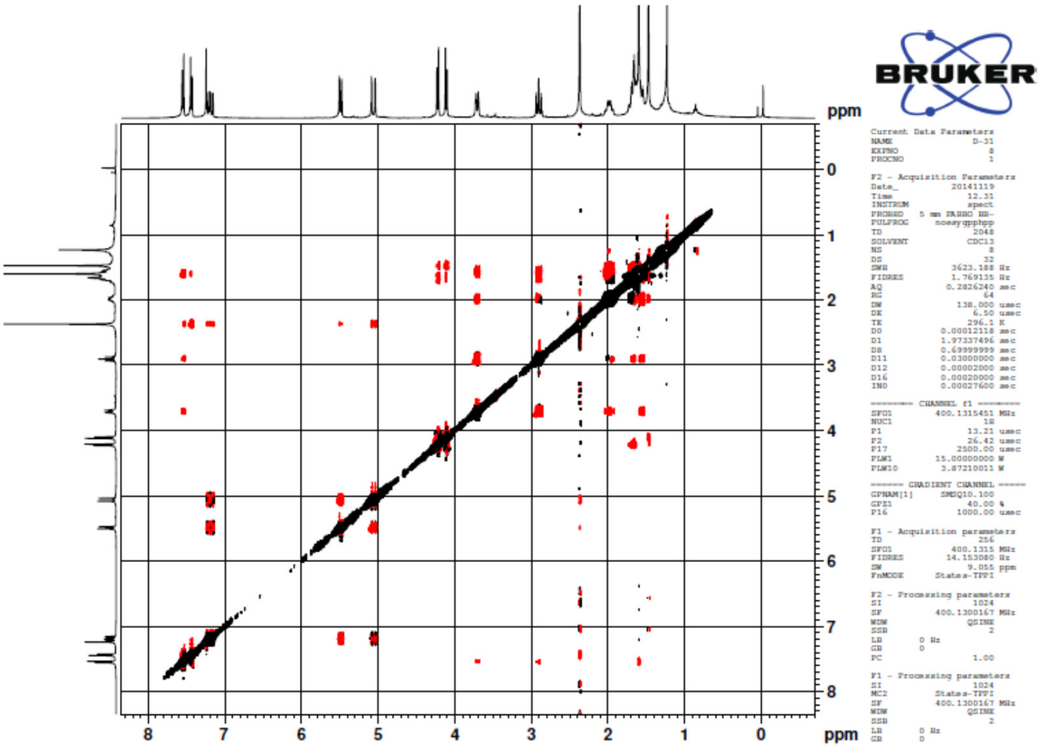

Figure S7. NOESY spectrum of **1**.

LIST: h150021-c1 29-Jan-15 Elapse: 05:50.7 28
Samp: D-31 Start : 13:40:49 29
Comm: Finnigan/MAT95//70eV/R:10000
Mode: EI +VE +LMR BSCAN (EXP) UP HR NRM Study : S/N: PT200712-01-01
Oper: SIMM.CAS Client: S/N: PT001263 Inlet :
Limt: (0) . .
: (413) C22.H100.O3
Peak: 1000.00 mmu R+D: -2.0 > 60.0
Data: CMASS : converted

Mass		Intensity (52607)	%RA	%RIC	Delta (mmu)	R+D	Composition
76.03173	*	59751	3.41	0.26	-0.4	5.0	C6.H4
89.03838	*	81660	4.66	0.35	0.7	5.5	C7.H5
111.0316	*	62668	3.57	0.27			
115.0535	*	169011	9.64	0.73	1.3	6.5	C9.H7
118.0417	*	83438	4.76	0.36	0.2	6.0	C8.H6.O
127.0526	*	60747	3.46	0.26	2.2	7.5	C10.H7
128.0623	*	96598	5.51	0.42	0.3	7.0	C10.H8
139.0546	*	61387	3.50	0.26	0.2	8.5	C11.H7
141.0699	*	67789	3.87	0.29	0.5	7.5	C11.H9
152.0615	*	190280	10.85	0.82	1.1	9.0	C12.H8
153.0699	*	139776	7.97	0.60	0.5	8.5	C12.H9
165.0708	*	342433	19.53	1.48	-0.4	9.5	C13.H9
166.0758	*	113670	6.48	0.49	2.5	9.0	C13.H10
167.0847	*	55341	3.16	0.24	1.4	8.5	C13.H11
178.0780	*	134939	7.70	0.58	0.3	10.0	C14.H10
179.0843	*	109331	6.23	0.47	1.7	9.5	C14.H11
181.1009	*	56621	3.23	0.24	0.8	8.5	C14.H13
189.0694	*	63521	3.62	0.27	1.0	11.5	C15.H9
195.0800	*	53491	3.05	0.23	1.0	9.5	C14.H11.O
197.0951	*	56052	3.20	0.24	1.5	8.5	C14.H13.O
209.0961	*	196682	11.22	0.85	0.6	9.5	C15.H13.O
211.0748	*	107552	6.13	0.46	1.1	9.5	C14.H11.O2
223.0742	*	57475	3.28	0.25	1.7	10.5	C15.H11.O2
223.1113	*	135365	7.72	0.58	1.0	9.5	C16.H15.O
224.1186	*	722425	41.20	3.11	1.5	9.0	C16.H16.O
225.0900	*	153220	8.74	0.66	1.5	9.5	C15.H13.O2
225.1220	*	116515	6.64	0.50			
235.0750	*	116160	6.62	0.50	0.9	11.5	C16.H11.O2
236.0833	*	300394	17.13	1.29	0.4	11.0	C16.H12.O2
237.0905	*	306796	17.50	1.32	1.0	10.5	C16.H13.O2
237.1272	*	55127	3.14	0.24	0.7	9.5	C17.H17.O
238.0973	*	95887	5.47	0.41	2.1	10.0	C16.H14.O2
249.0908	*	95602	5.45	0.41	0.8	11.5	C17.H13.O2
250.0985	*	88560	5.05	0.38	0.9	11.0	C17.H14.O2
251.1065	*	266606	15.20	1.15	0.7	10.5	C17.H15.O2
252.1154	*	931414	53.12	4.02	-0.3	10.0	C17.H16.O2
253.0860	*	188217	10.73	0.81	0.4	10.5	C16.H13.O3
253.1202	*	220511	12.57	0.95	2.6	9.5	C17.H17.O2
254.1268	*	55697	3.18	0.24			
265.1219	*	65086	3.71	0.28	1.0	10.5	C18.H17.O2
267.1400	*	423525	24.15	1.83	-1.5	9.5	C18.H19.O2
268.1439	*	66082	3.77	0.28	2.5	9.0	C18.H20.O2
280.1102	*	158982	9.07	0.69	-0.2	11.0	C18.H16.O3
280.1475	*	225704	12.87	0.97	-1.2	10.0	C19.H20.O2
294.1265	*	80380	4.58	0.35	-0.9	11.0	C19.H18.O3
295.1339	*	1753568	100.00	7.56	-0.5	10.5	C19.H19.O3
296.1369	*	359505	20.50	1.55			
297.1422	*	52709	3.01	0.23			
308.1408	*	106557	6.08	0.46	0.4	11.0	C20.H20.O3
309.1462	*	72128	4.11	0.31	2.9	10.5	C20.H21.O3
310.1570	*	1022606	58.32	4.41	-0.1	10.0	C20.H22.O3
311.1600	*	222788	12.70	0.96			

Figure S8. HREIMS spectrum of **1**.

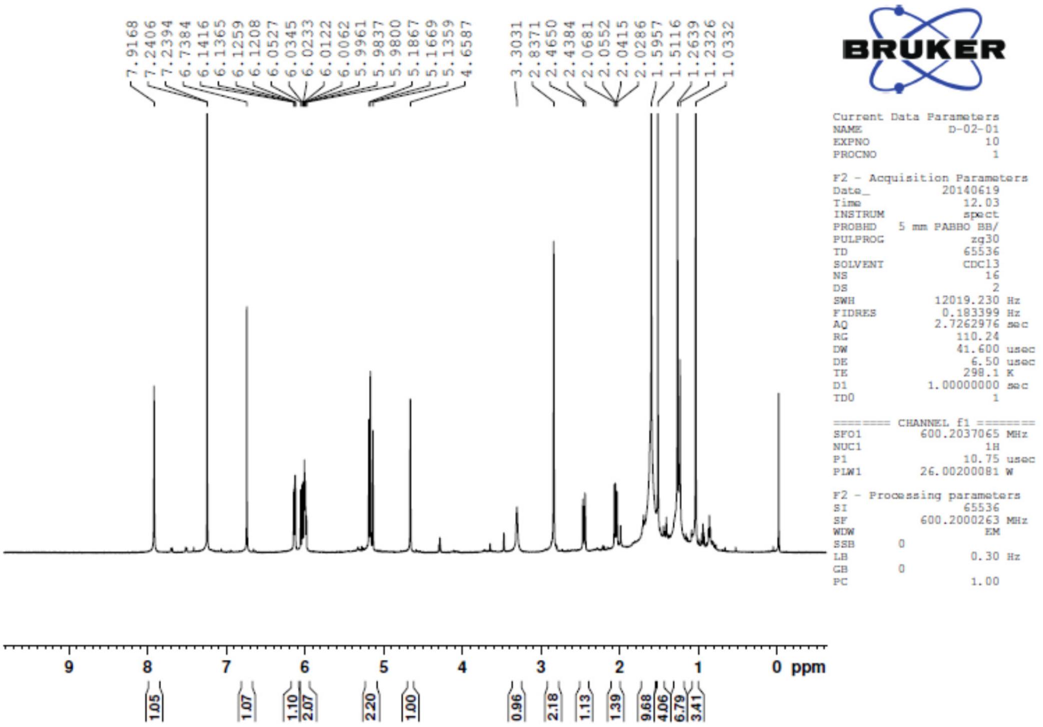

Figure S9. ^{1}H NMR ($CDCl_3$, 600 MHZ) spectrum of **2**.

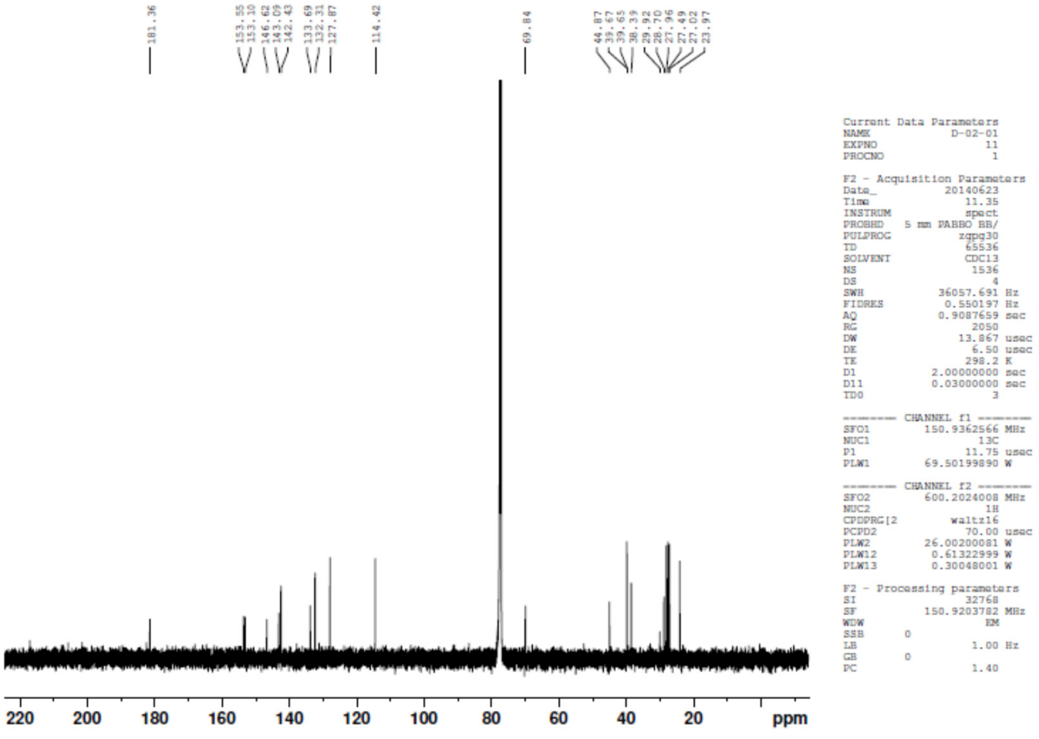

Figure S10. ^{13}C NMR ($CDCl_3$, 150 MHZ) spectra of **2**.

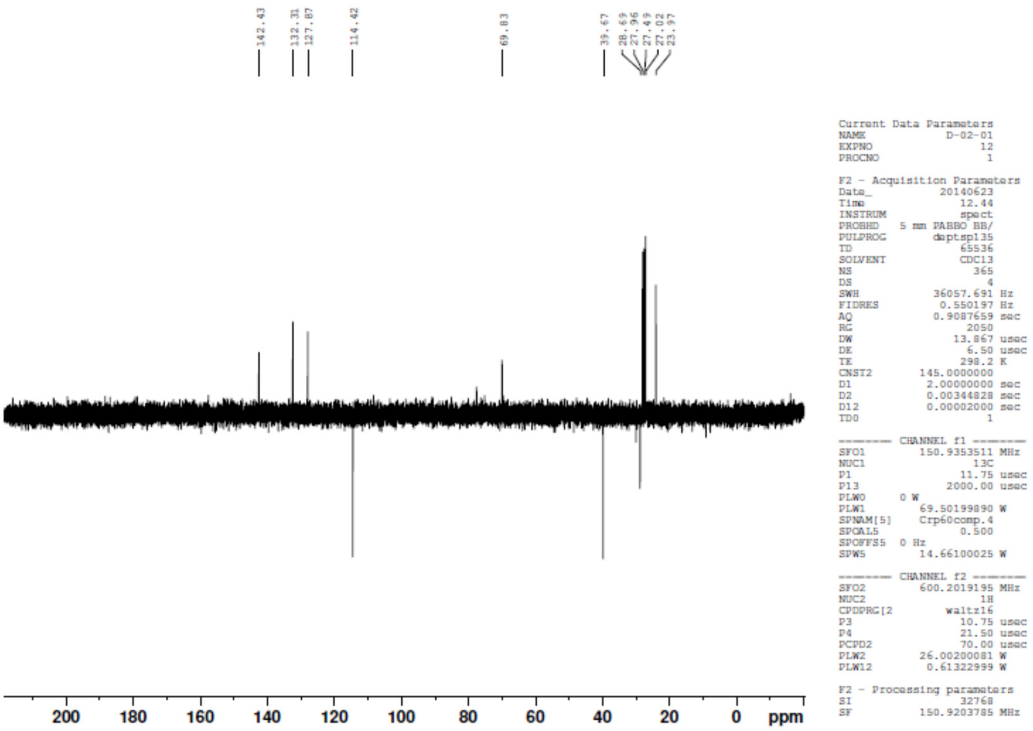

Figure S11. DEPT (CDCl3, 150 MHZ) spectrum of **2**.

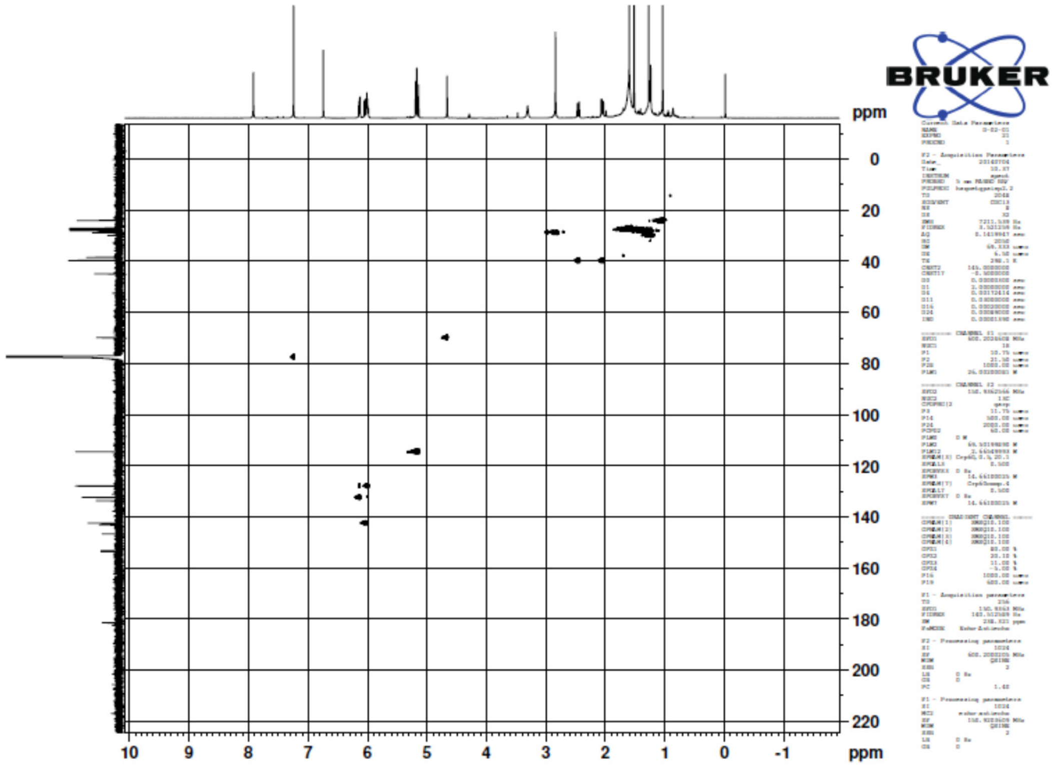

Figure S12. HSQC spectrum of **2**.

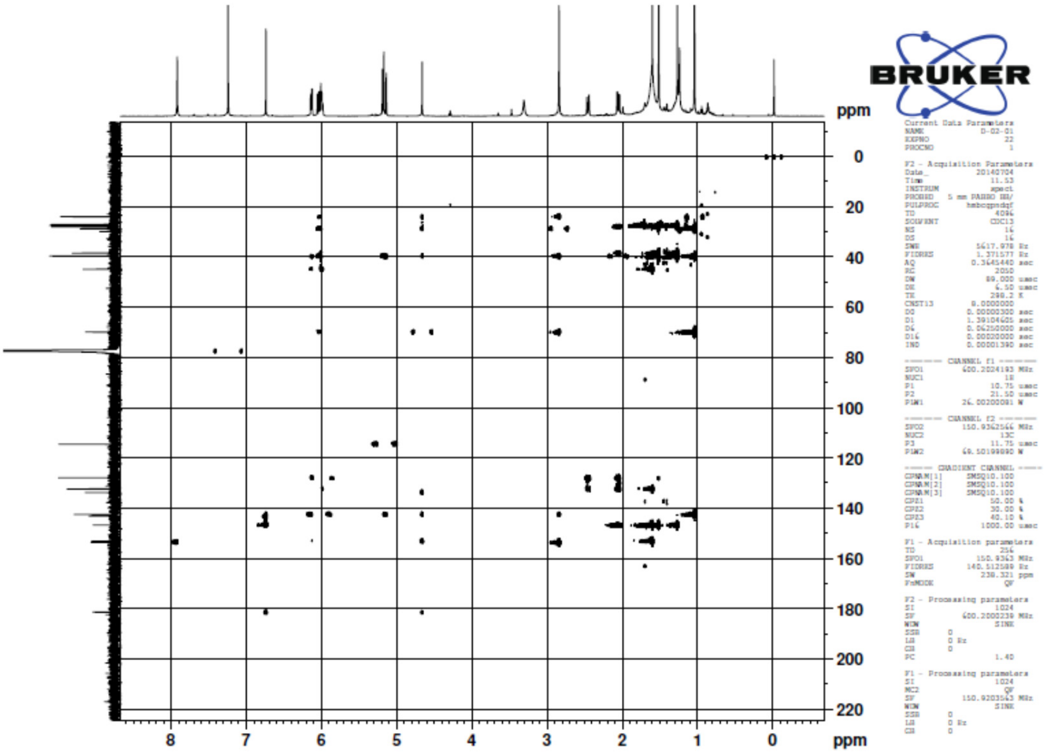

Figure S13. HMBC spectrum of **2**.

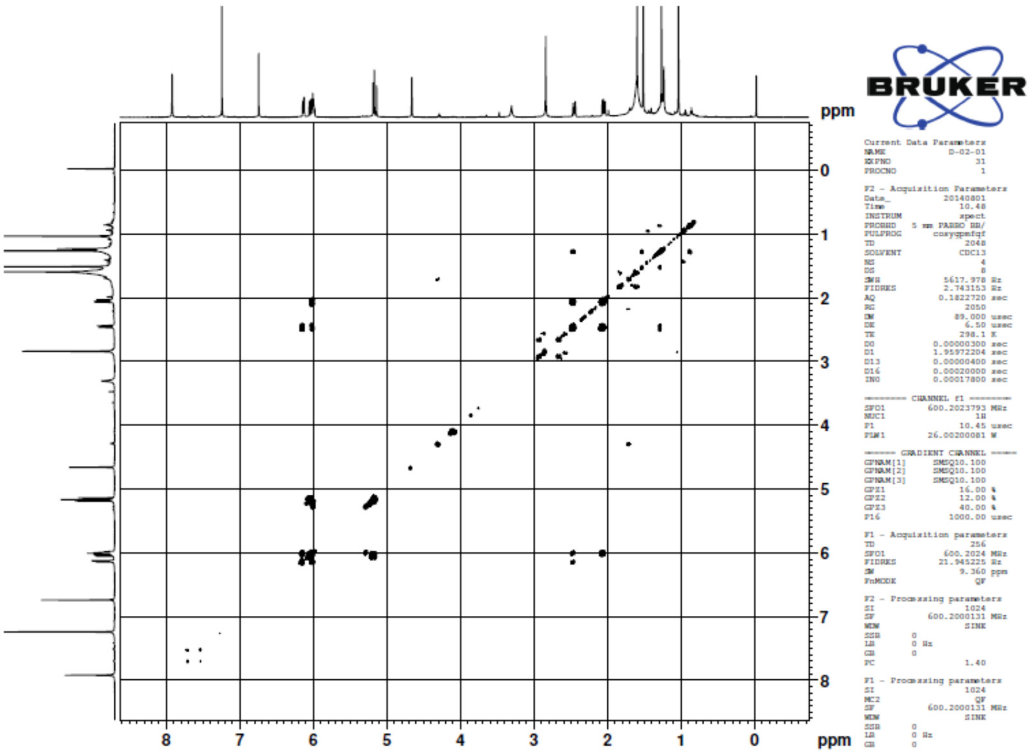

Figure S14. COSY spectrum of **2**.

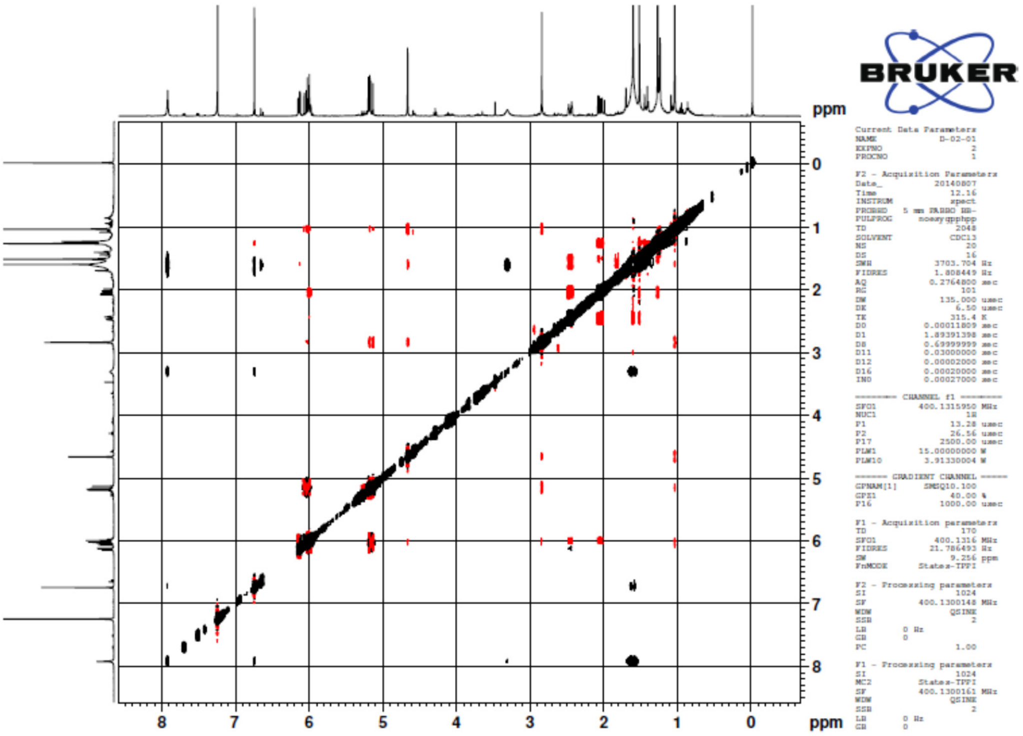

Figure S15. NOESY spectrum of **2**.

```
LIST:  h140583-cl                             22-Aug-14   Elapse:  05:23.8      26
Samp:  D-0201                                             Start :  14:36:39     32
Comm:  Finnigan/MAT95//70eV/R:10000
Mode:  EI +VE +LMR  BSCAN (EXP) UP HR NRM                 Study :  S/N: PT200712-01-01
Oper:  WANG_J@SIMM.AC.CN   Client:  S/N: PT001263         Inlet :
Limt:  (    0)
    :  ( 443 ) C22.H100.N.O4
Peak:  1000.00  mmu    R+D:  -2.0 > 60.0
Data:  CMASS : converted
```

Mass	Intensity (24376)	%RA	%RIC	Delta (mmu)	R+D	Composition
71.08585 *	25999	11.73	0.11	0.2	0.5	C5.H11
73.02899 *	28706	12.95	0.12	0.0	1.5	C3.H5.O2
77.03950 *	61900	27.93	0.26	-0.4	4.5	C6.H5
79.05518 *	39533	17.84	0.17	-0.4	3.5	C6.H7
81.06956 *	32410	14.63	0.14	0.9	2.5	C6.H9
83.08472 *	24432	11.03	0.10	1.4	1.5	C6.H11
85.06452 *	33835	15.27	0.14	0.8	1.5	C5.H9.O
91.05419 *	67528	30.47	0.28	0.6	4.5	C7.H7
105.0345 *	34120	15.40	0.14	-0.4	5.5	C7.H5.O
111.9514 *	25287	11.41	0.11			
115.0317 *	63752	28.77	0.27			
127.0529 *	31769	14.34	0.13	1.9	7.5	C10.H7
128.0609 *	61971	27.96	0.26	1.7	7.0	C10.H8
129.0682 *	31840	14.37	0.13	2.2	6.5	C10.H9
141.0684 *	28777	12.99	0.12	2.0	7.5	C11.H9
143.0860 *	25643	11.57	0.11	0.1	6.5	C11.H11
149.0228 *	133275	60.14	0.56	1.1	6.5	C8.H5.O3
152.0612 *	31057	14.01	0.13	1.4	9.0	C12.H8
153.0656 *	27353	12.34	0.11			
154.0681 *	25714	11.60	0.11	-2.4	8.5	C11.H8.N
165.0683 *	43237	19.51	0.18	2.2	9.5	C13.H9
165.0906 *	40388	18.23	0.17	0.9	4.5	C10.H13.O2
171.0798 *	36969	16.68	0.15	1.2	7.5	C12.H11.O
199.0729 *	26712	12.05	0.11	3.0	8.5	C13.H11.O2
223.1053 *	24646	11.12	0.10			
224.1096 *	25429	11.48	0.11	-2.1	9.5	C15.H14.N.O
232.1096 *	32196	14.53	0.13	0.3	7.0	C14.H16.O3
236.1444 *	40388	18.23	0.17	-0.5	9.5	C17.H18.N
250.1229 *	32980	14.88	0.14	0.2	10.5	C17.H16.N.O
				-2.4	6.0	C14.H18.O4
252.1027 *	42026	18.96	0.18	-0.2	10.5	C16.H14.N.O2
253.1139 *	34974	15.78	0.15			
254.1188 *	30914	13.95	0.13	-0.7	9.5	C16.H16.N.O2
258.1109 *	34547	15.59	0.14	2.1	8.5	C15.H16.N.O3
264.1371 *	45517	20.54	0.19	-0.9	6.0	C15.H20.O4
				1.8	10.5	C18.H18.N.O
270.1142 *	91960	41.50	0.39	-1.2	9.5	C16.H16.N.O3
278.1188 *	28991	13.08	0.12	-0.7	11.5	C18.H16.N.O2
280.1699 *	79281	35.78	0.33	0.3	9.5	C19.H22.N.O
				-2.4	5.0	C16.H24.O4
282.1128 *	52711	23.79	0.22	0.2	10.5	C17.H16.N.O3
282.1511 *	44021	19.86	0.18	-1.7	9.5	C18.H20.N.O2
292.1342 *	35544	16.04	0.15	-0.5	11.5	C19.H18.N.O2
293.1462 *	33835	15.27	0.14			
294.1495 *	25287	11.41	0.11	-0.1	10.5	C19.H20.N.O2
297.1704 *	53780	24.27	0.23	2.5	9.0	C19.H23.N.O2
308.1633 *	212414	95.85	0.89	1.8	10.5	C20.H22.N.O2
309.1664 *	61117	27.58	0.26			
310.1475 *	103571	46.74	0.43			
314.1766 *	47796	21.57	0.20	-1.0	8.5	C19.H24.N.O3
324.1609 *	31270	14.11	0.13	-0.9	10.5	C20.H22.N.O3
325.1663 *	221603	100.00	0.93	1.5	10.0	C20.H23.N.O3
326.1719 *	148020	66.80	0.62			
327.1762 *	30416	13.73	0.13			
328.1592 *	28421	12.83	0.12			
343.1780 *	53281	24.04	0.22	0.3	9.0	C20.H25.N.O4

Figure S16. HREIMS spectrum of **2**.

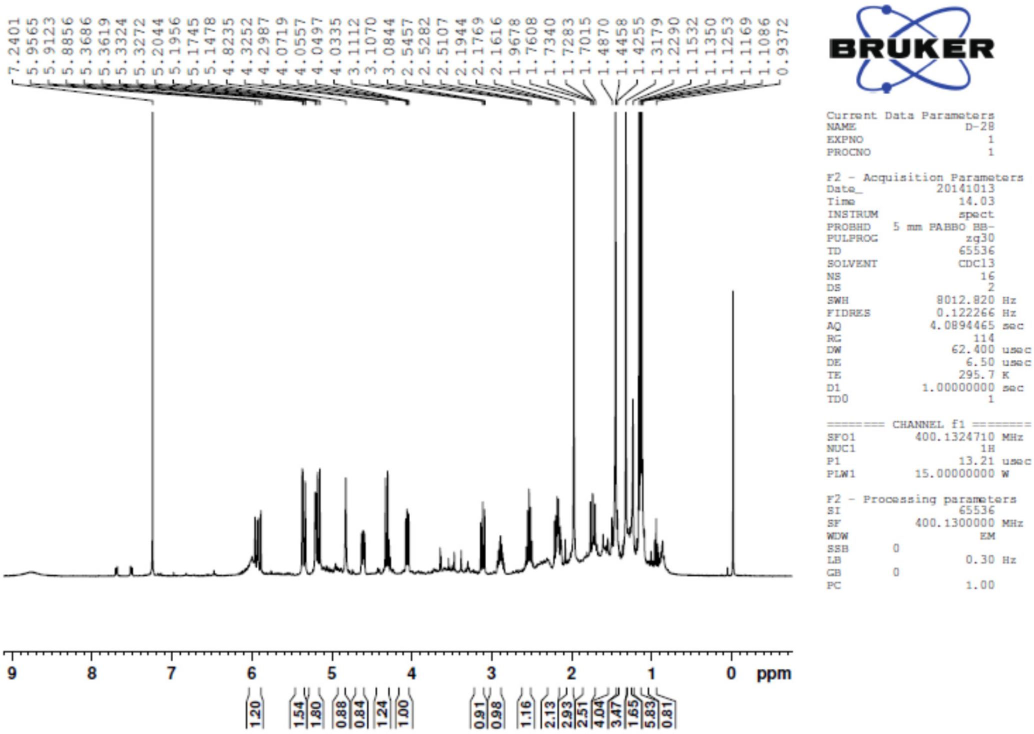

Figure S17. ^{1}H NMR ($CDCl_3$, 400 MHZ) spectrum of **3**.

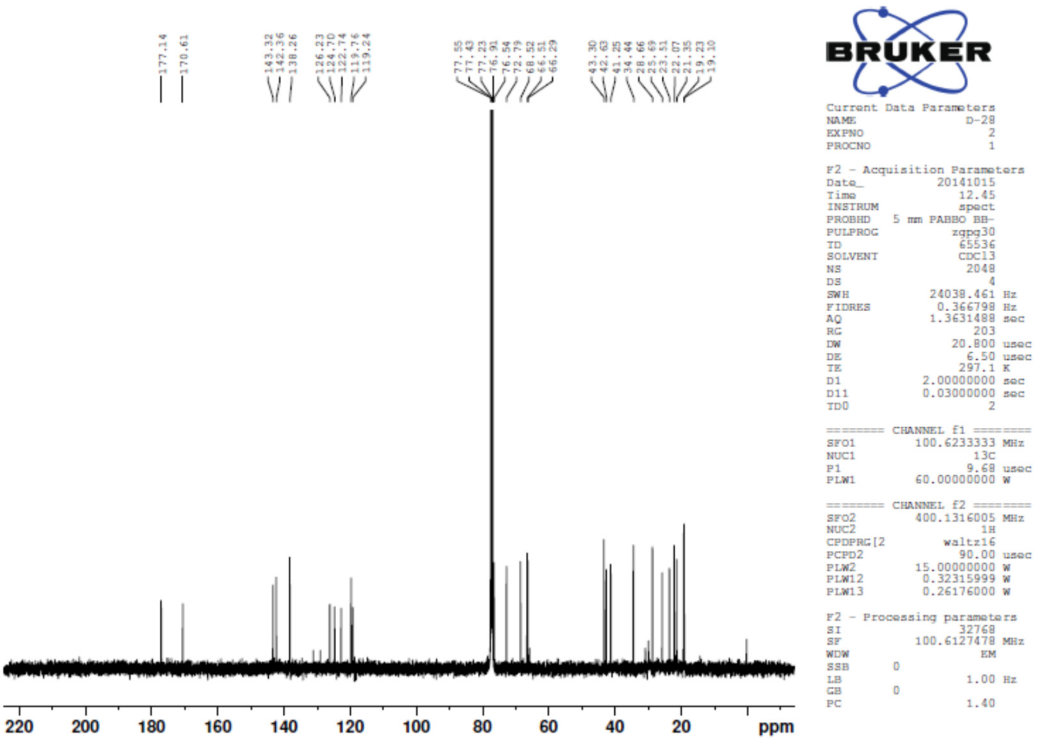

Figure S18. ^{13}C NMR ($CDCl_3$, 100 MHZ) spectra of **3**.

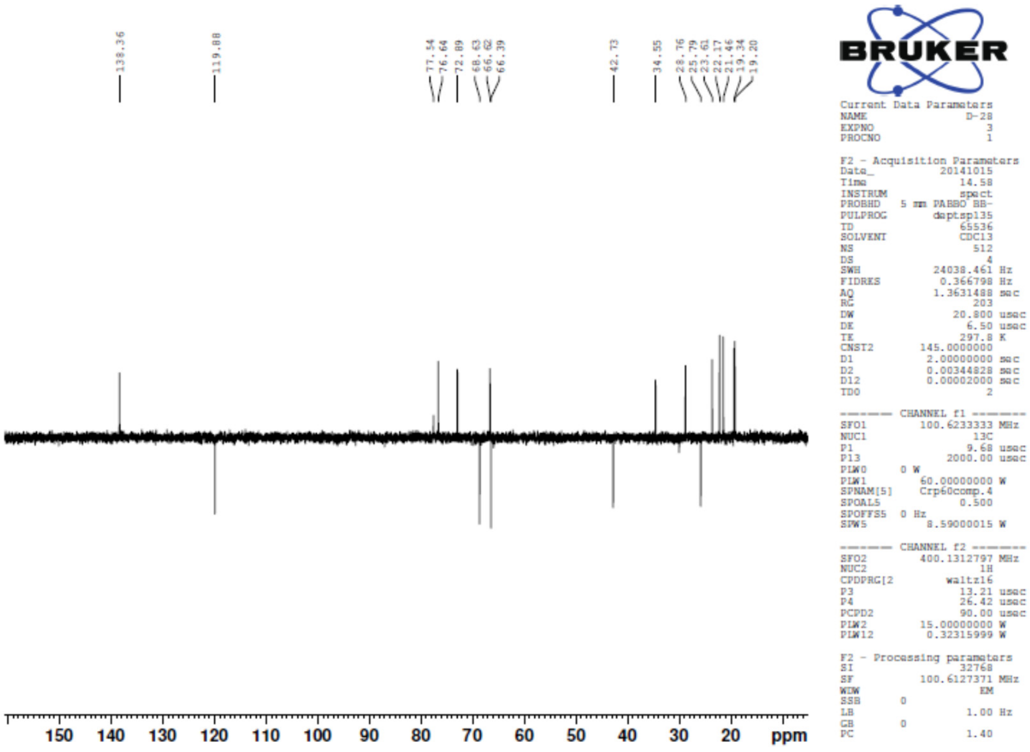

Figure S19. DEPT ($CDCl_3$, 100 MHZ) spectrum of **3**.

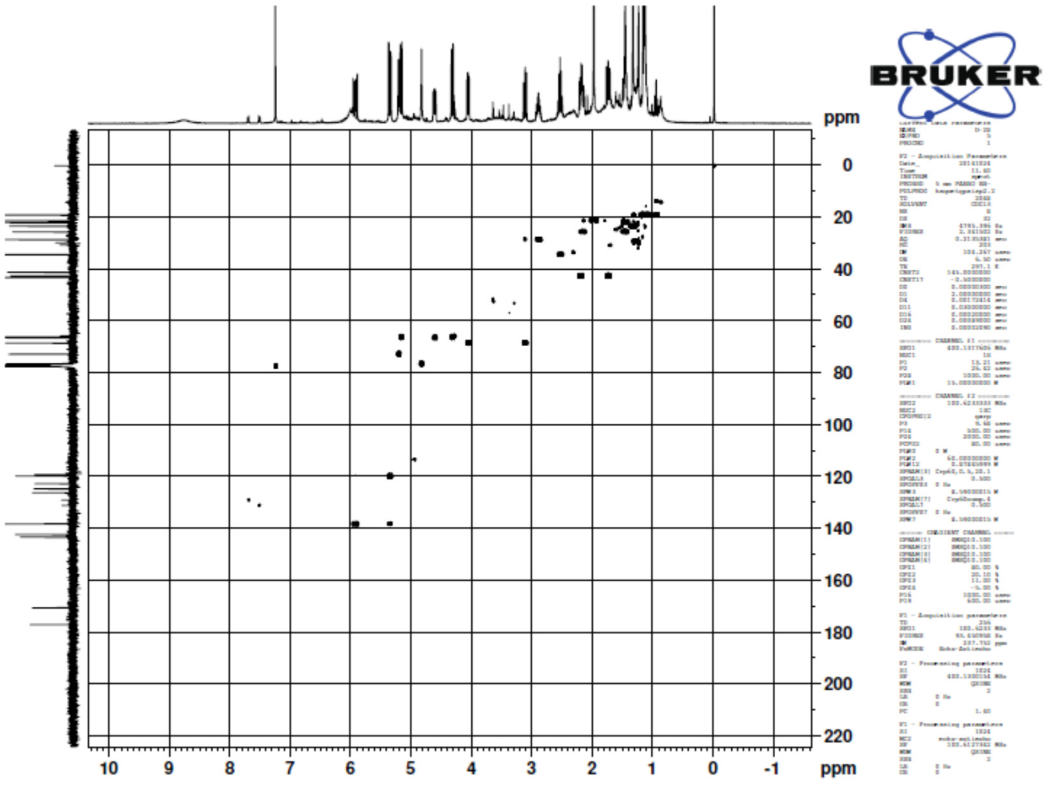

Figure S20. HSQC spectrum of **3**.

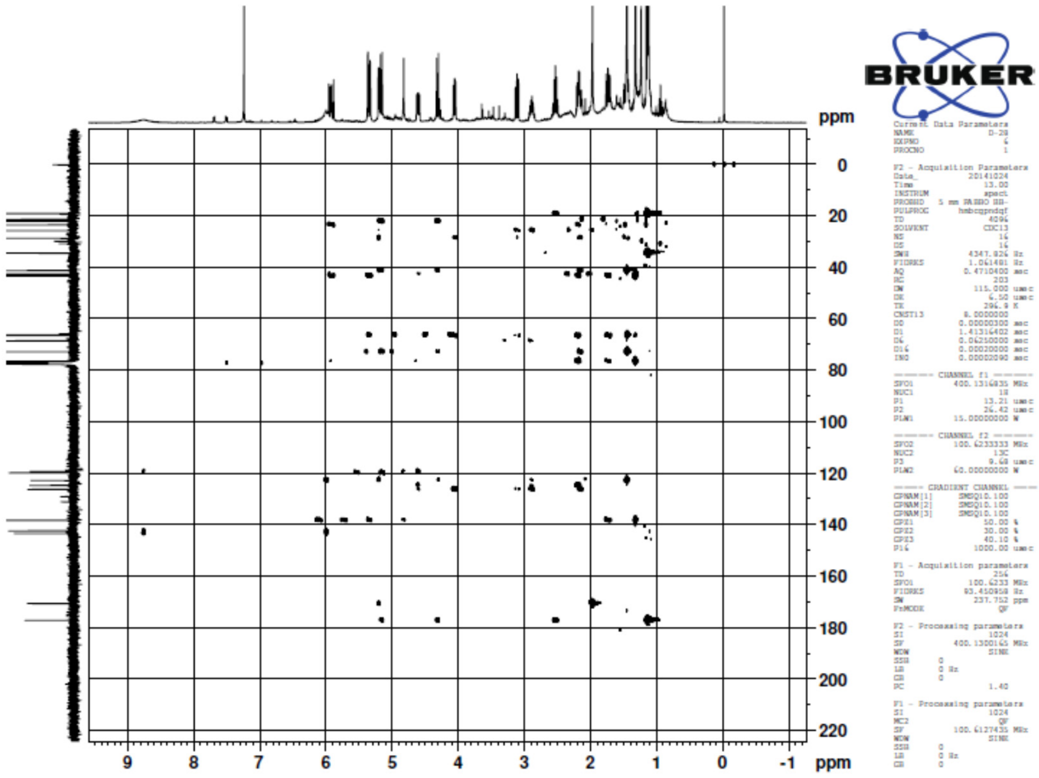

Figure S21. HMBC spectrum of **3**.

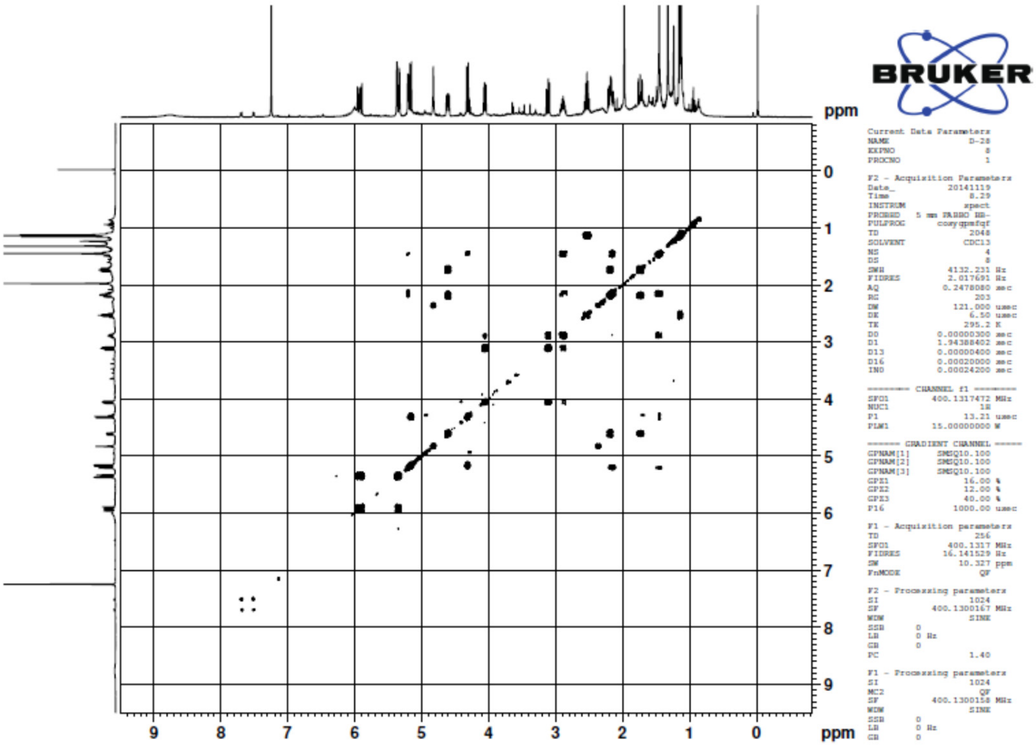

Figure S22. COSY spectrum of **3**.

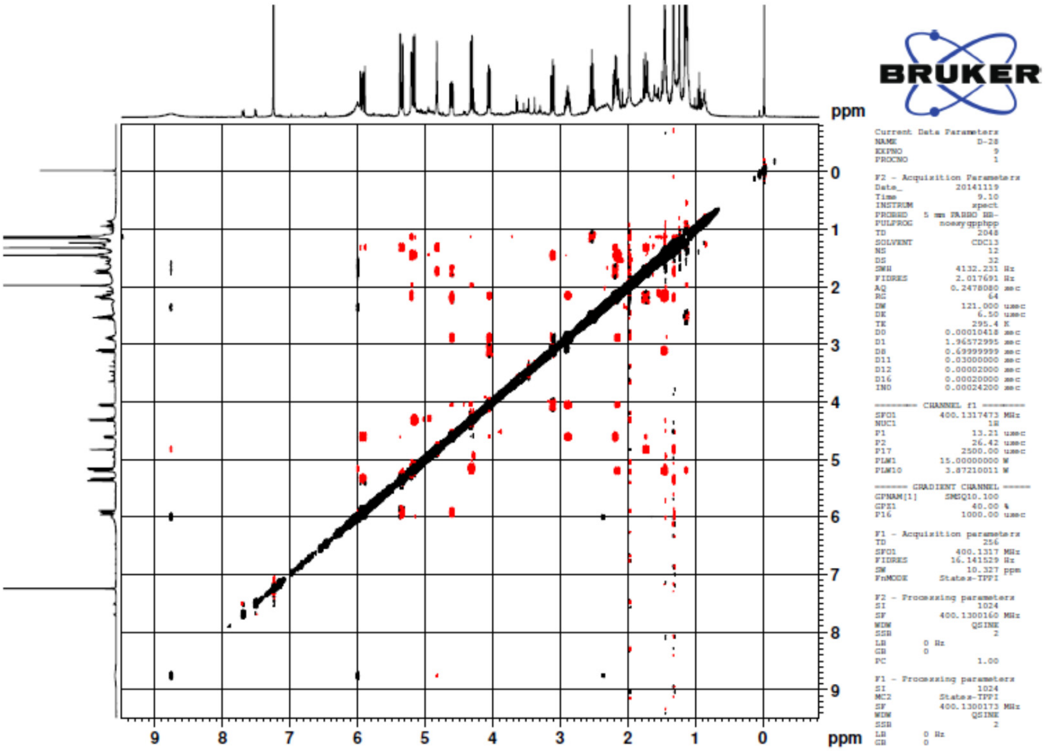

Figure S23. NOESY spectrum of **3**.

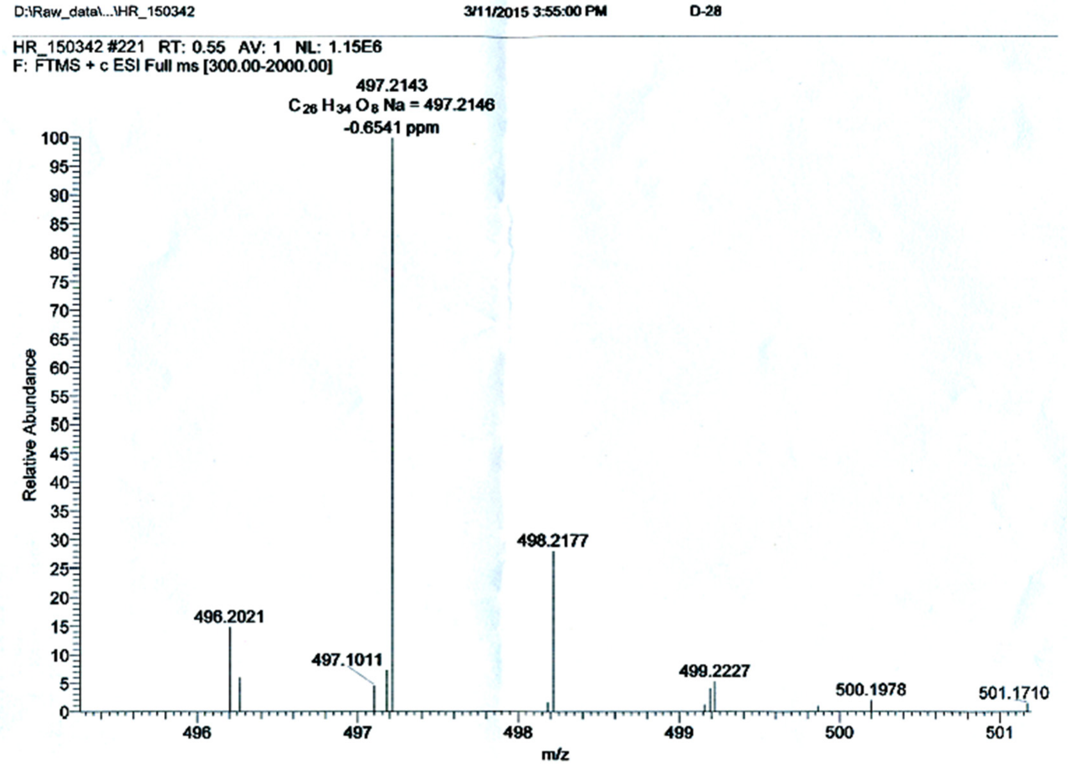

Figure S24. HRESIMS spectrum of **3**.
